# The dynamin Vps1 mediates Atg9 transport to the sites of autophagosome formation

**DOI:** 10.1016/j.jbc.2023.104712

**Published:** 2023-04-14

**Authors:** Henning Arlt, Babu Raman, Yasmina Filali-Mouncef, Yan Hu, Alexandre Leytens, Ralph Hardenberg, Rodrigo Guimarães, Franziska Kriegenburg, Muriel Mari, Iwona I. Smaczynska-de Rooij, Kathryn R. Ayscough, Jörn Dengjel, Christian Ungermann, Fulvio Reggiori

**Affiliations:** 1 Department of Biomedical Sciences of Cells and Systems, University of Groningen, University Medical Center Groningen, Groningen, The Netherlands; 2 University of Osnabrück, Department of Biology/Chemistry, Biochemistry section, Osnabrück, Germany; 3 University of Osnabrück, Center of Cellular Nanoanalytics (CellNanOs), Osnabrück, Germany; 4 Department of Biomedicine, Aarhus University, Aarhus, Denmark; 5 Department of Biology, University of Fribourg, Fribourg, Switzerland; 6 Department of Biomedical Sciences, University of Sheffield, Sheffield, United Kingdom; 7 Aarhus Institute of Advanced Studies (AIAS), Aarhus University, Aarhus, Denmark

**Keywords:** autophagy, autophagosome, atg9, dynamin, vps1, sorting nexin, SNX, retromer, PAS, phagophore

## Abstract

Autophagy is a key process in eukaryotes to maintain cellular homeostasis by delivering cellular components to lysosomes/vacuoles for degradation and reuse of the resulting metabolites. Membrane rearrangements and trafficking events are mediated by the core machinery of autophagy-related (Atg) proteins, which carry out a variety of functions. How Atg9, a lipid scramblase and the only conserved transmembrane protein within this core Atg machinery, is trafficked during autophagy remained largely unclear. Here, we addressed this question in yeast *Saccharomyces cerevisiae* and found that retromer complex and dynamin Vps1 mutants alter Atg9 subcellular distribution and severely impair the autophagic flux by affecting two separate autophagy steps. We provide evidence that Vps1 interacts with Atg9 at Atg9 reservoirs. In the absence of Vps1, Atg9 fails to reach the sites of autophagosome formation, and this results in an autophagy defect. The function of Vps1 in autophagy requires its GTPase activity. Moreover, Vps1 point mutants associated with human diseases such as microcytic anemia and Charcot-Marie-Tooth are unable to sustain autophagy and affect Atg9 trafficking. Together, our data provide novel insights on the role of dynamins in Atg9 trafficking and suggest that a defect in this autophagy step could contribute to severe human pathologies.

Macroautophagy (hereafter autophagy) is a transport pathway highly conserved in eukaryotes involved in the recycling of cellular components upon stress or starvation conditions but also in the elimination of specific unwanted structures to maintain cell homeostasis. During autophagy, targeted intracellular material is sequestered into newly formed double-membrane vesicles called autophagosomes, which eventually fuse with the vacuole in yeast and plants, or lysosomes in mammals, to degrade their contents (1). This process is mediated by the core Atg machinery, which is composed of approximately 20 proteins that are subdivided in six functional groups (1). Most of the core Atg proteins are peripheral membrane-associated proteins that are recruited from the cytosol to autophagosomal intermediate structures to carry out specific functions (1). An exception is the multispanning transmembrane protein Atg9, which is a key organizing component of the core Atg machinery and implicated in the initial membrane delivery to the forming autophagosomes (2, 3, 4, 5, 6). Moreover, it has recently been shown that Atg9 and ATG9A form trimers with a lipid scramblase activity (7, 8), which is important for the supply of lipids to the nascent autophagosomes by Atg2 lipid transfer proteins (9). In yeast, Atg9 is delivered after synthesis in the endoplasmic reticulum to a post-Golgi compartment designed as the Atg9 reservoirs or Atg9 vesicles (2, 3). From there, Atg9-containing vesicles reach the phagophore assembly site or pre-autophagosomal structure (PAS) through an Atg11- or Atg17-dependent mechanism, differing on the cues triggering the induction of autophagosome formation (10, 11) (Fig. 1*A*). The PAS is a perivacuolar site also proximal to the ER, where the Atg machinery assembles to generate an autophagosome (12, 13, 14, 15). After autophagosome completion, Atg9 is recycled during or after autophagosome-vacuole fusion, since it is detected on complete autophagosomes but not on the vacuole membrane (2, 3, 6, 16, 17).

**Figure 1:**
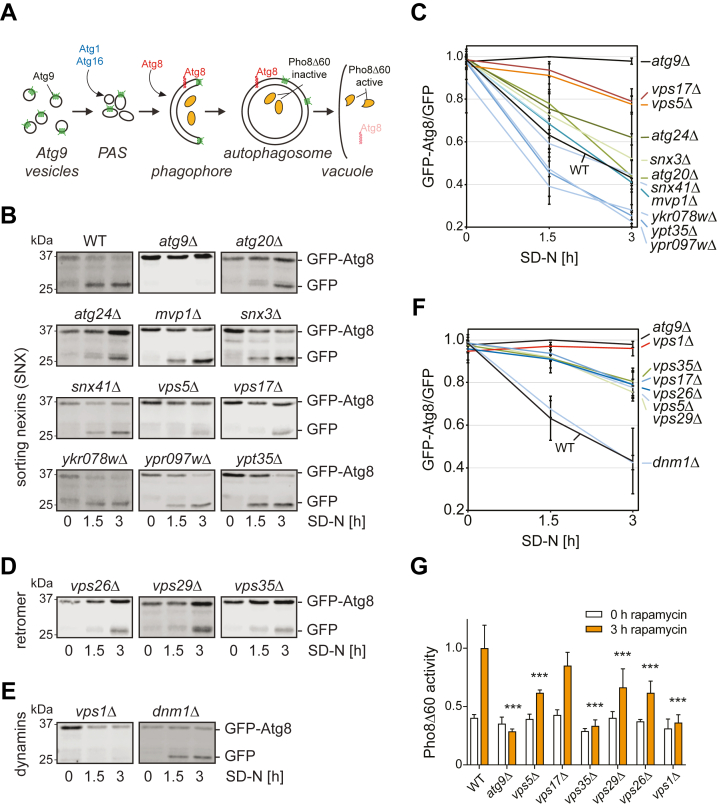
**Cells lacking subunits of the retromer or Vps1 have an autophagy defect.***A*, schematic model of yeast autophagy. Major Atg proteins and cargo molecules examined in this study are indicated. *B*–*F*, the indicated strains (see Table 3) were transformed with the pCuGFPAUT7(416) plasmid, which expresses the GFP-Atg8 chimera, before grown in SMD medium lacking uracil and subsequently starved for nitrogen in SD-N medium for 3 h. Aliquots of cells were collected 0, 1.5, and 3 h after the medium change, and proteins were analyzed by western blots using anti-GFP antibodies (*B*, *D*, and *E*). Three independent experiments were quantified, and graphs indicate mean values ± SD (*C* and *F*). *G*, the Pho8Δ60 assay was performed in a WT (YTS159) and the same strain background lacking *ATG9* (FRY244), *VPS1* (HAY164), *VPS5* (HAY161), *VPS17* (HAY162), *VPS26* (HAY245), *VPS29* (HAY205), and *VPS35* (HAY163). Pho8Δ60 activity was measured before and after the addition of rapamycin for 3 h to cells grown in YPD medium. Data represent the average of four independent experiments expressed relatively to the WT treated with rapamycin for 3 h. The graphs indicate mean values ± SD, and significant differences of the rapamycin-treated samples with the WT are indicated. ∗∗∗*p* < 0.0001.

Many trafficking events in the endomembrane system require a conserved set of machineries including proteins of the dynamin and sorting nexin protein families. Sorting nexins (SNX) are recruited to membranes *via* their Phox-homology (PX) domains, which bind to phosphoinositides. Some of the members of this protein family are able to deform or sense membrane curvature through a BAR domain (18). SNX can act in concert with larger protein complexes, for example, the SNX Vps5 and Vps17 interact with the retromer complex, to facilitate the retrieval of transmembrane proteins from endosomes to the Golgi (18). Dynamins, on the other hand, are GTPases that oligomerize and are implicated in membrane remodeling, in membrane fission events necessary to generate vesicles or separate organelles (19). Importantly, mutation in these proteins leads to severe diseases. For example, mutations in DYNAMIN1 (DNM1) cause epileptic encephalopathy, whereas DYNAMIN2 (DNM2) is found mutated in microcytic anemia, centronuclear myopathy, and Charcot-Marie-Tooth disease (20, 21).

A largely unresolved key question in autophagy is how Atg9 is transported to the site of autophagosome biogenesis. As yeast Atg9 and mammalian ATG9A traffics through multiple compartments, including the Golgi, endosomes, and the plasma membrane, multiple membrane transport components regulate their trafficking. These include the adapter protein complexes AP2 and AP4, the TBC1 domain family members TBC1D5 and TBC1D14, the retromer, and the TRAPPIII complex (*e.g.*, (22, 23, 24, 25, 26, 27, 28, 29, 30)). Most of these factors are important in sorting and maintaining Atg9 and ATG9A in post-Golgi reservoirs. Recycling endosomes appear to be key in sorting membranes necessary for the phagophore nucleation and/or expansion (30, 31). In this context, initial release of mammalian ATG9A-positive membranes from recycling endosomes during autophagy requires SNX18 and the BAR domain–containing BIF1, in concert with DNM2 (32, 33). However, the mechanisms of downstream trafficking events after vesicle release remain unclear.

Here, we address this question using yeast *Saccharomyces cerevisiae*. We show that the SNX Vps5 and Vps17, the retromer complex, and the dynamin Vps1 are involved in autophagy, however, in two different pathways. Moreover, we provide evidence for a direct link between the yeast dynamin Vps1 and Atg9 transport to the PAS. In the absence of Vps1, Atg9 trafficking to the PAS is impaired, causing a severe defect in autophagic flux. This phenotype can be rescued by reintroducing WT *VPS1*, but not GTPase defective mutants. Moreover, a few Vps1 variants associated with microcytic anemia and Charcot-Marie-Tooth also impair Atg9 transport to the PAS and autophagy progression. This result infers that an impairment of autophagy could be a contributing etiological factor of these diseases.

## Results

### Retromer and Vps1 are essential for autophagy

To acquire new information about the membrane rearrangement processes underlying autophagosome formation, we decided to explore whether proteins known to reshape lipid bilayers are involved in autophagy. One class of factors that deform membranes during transmembrane protein sorting events are the SNX proteins (18). Although two members of this protein family, Atg20 and Atg24, are required for the cytoplasm-to-vacuole targeting (Cvt) pathway (34), none of them have been shown to be involved in autophagy in yeast.

To determine whether one or more SNX proteins are involved in yeast autophagy, we analyzed the progression of bulk autophagy in the corresponding KO mutants using the GFP-Atg8 processing assay (35). In WT cells, the GFP-Atg8 chimera is delivered *via* autophagy to the vacuole where Atg8 is degraded but GFP moiety persists since it is resistant to vacuolar proteases. As a result, accumulation of free GFP over time can be followed by Western blot, allowing to assess autophagy progression upon its induction, for example, by nitrogen starvation in SD-N medium (Fig. 1*B*). In contrast to WT cells in which GFP-Atg8 is almost completely degraded into free GFP after 3 h of nitrogen deprivation, mutants with a complete block in autophagy like *atg9Δ* show no accumulation of free GFP (Fig. 1, *B* and *C*). Using this method, we found that among all the tested SNX deletion mutants (*atg20Δ, atg24Δ, mvp1Δ, snx3Δ, snx41Δ, vps5Δ, vps17Δ, ykr078wΔ*, *ypr097wΔ*, and *ypt35Δ*), only *vps5Δ* and *vps17Δ* mutants showed a clear reduction in the accumulation of free GFP after 3 h of nitrogen starvation (Fig. 1, *B* and *C*). Pairwise combination of the examined SNX deletions displayed no further GFP-Atg8 processing defect (not shown), indicating no functional redundancy between SNX proteins in yeast autophagy.

Vps5 and Vps17 form a heterodimer and interact with the retromer. Therefore, we wondered if KO strains lacking retromer subunits show similar defects. In agreement with a previous study (36), all KO mutants lacking a subunit of the retromer, Vps26, Vps29, or Vps35, displayed a severe impairment in GFP-Atg8 processing indicating a defect in autophagy (Fig. 1, *D* and *F*). Retromer functionally cooperates with the dynamin Vps1 (37, 38). Therefore, we asked whether dynamins might also be involved in autophagy and tested both yeast dynamin-like proteins, that is, Vps1 and Dnm1, for their role in autophagy using the GFP-Atg8 processing assay as well. Interestingly, the *vps1Δ* mutant showed a clear defect in GFP-Atg8 turnover, while *dnm1Δ* cells were indistinguishable from the WT (Fig. 1, *E* and *F*). To validate our results, we tested the relevance of retromer and Vps1 in autophagy using a different method, that is, the Pho8Δ60 assay (35). Briefly, upon induction of autophagy, the Pho8Δ60 construct is delivered by autophagosomes into the vacuole lumen where it is processed into an activated form by resident proteases (Fig. 1*A*). Thus, after 3 h of treatment with rapamycin, a molecule inducing autophagy through the inhibition of the target of rapamycin (TOR) kinase (39), colorimetric enzymatic measurement of Pho8Δ60 activity in WT cells, but not in strains lacking key components of the autophagy machinery such as Atg9, allows to measure bulk autophagy induction (Fig. 1*G*). In agreement with the results obtained with the GFP-Atg8 processing assay, all analyzed mutants (*vps1Δ, vps5Δ, vps17Δ, vps26Δ*, *vps29Δ*, and *vps35Δ*), except for the *vps17Δ* knockout, showed a defect in autophagy (Fig. 1*G*). However, only *vps35Δ* and *vps1Δ* mutants displayed a reduction in Pho8Δ60 activity comparable to a strain with a complete block in autophagy, that is, the *atg9Δ* knockout (Fig. 1*G*). To show the direct involvement of these proteins in autophagy, in particular of Vps1, we transformed a plasmid carrying the thermosensitive allele *vps1-100* (40) into the Pho8Δ60 strain lacking *VPS1* and measured autophagy progression at both permissive and nonpermissive temperature. While cells starved for nitrogen at 24 °C showed autophagic activity, those transferred in the SD-N medium at 37 °C displayed a severe autophagy block (Fig. S1*A*). We concluded that the retromer and Vps1 are required in normal progression of bulk autophagy. For the rest of the study, we decided to focus on Vps1 and just one of the retromer subunits, Vps35.

### Retromer and *vps1* mutants affect different steps of autophagy

To gain insights into which step of autophagy the retromer and Vps1 are involved, we analyzed the localization of various protein markers for autophagosomal membranes in the absence of these factors over the course of a 3 h nitrogen starvation.

**Figure 2:**
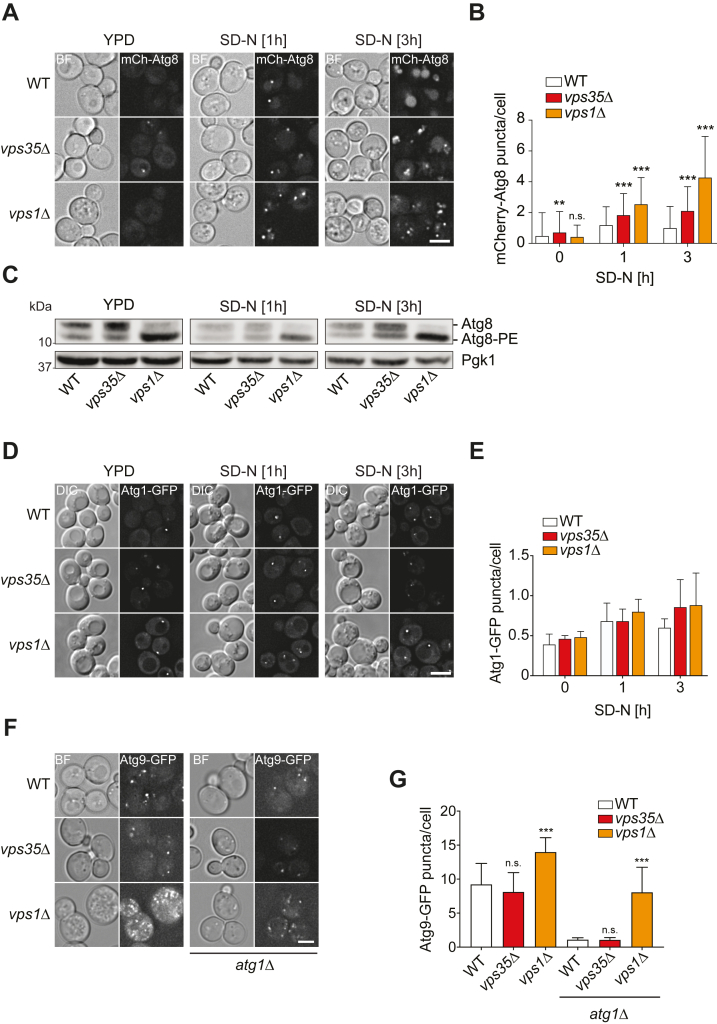
**Retromer and Vps1 mutants affect different autophagy steps.***A*, WT (HAY211), *vps35Δ* (HAY253), and *vps1Δ* (HAY296) cells expressing mCherry-V5-Atg8 were grown in YPD medium and then starved in SD-N medium. Cells were imaged by fluorescence microscopy and brightfield at the indicated times. Representative single z-focal planes are shown. *B*, quantification of the experiment shown in panel (*A*) by determining the average number of mCherry-V5-Atg8 puncta per cell from three independent experiments. Significant differences with the WT at each time point are indicated. ∗∗*p* < 0.0015; ∗∗∗*p* < 0.0001. *C*, WT (SEY6210), *vps35Δ* (HAY254), and *vps1Δ* (HAY294) cells were grown as in panel (*A*), harvested, and lysed with TCA, before Western blot analysis using an anti-Atg8 or anti-Pgk1 antibodies. Pgk1 served as a loading control. *D*, WT (RSGY044), *vps35Δ* (RSGY046), and *vps1Δ* (RSGY047) expressing endogenous *ATG1-GFP* were treated as in (*A*) and imaged by fluorescence microscopy. *E*, quantification of the experiment shown in panel (*D*) by determining the number of Atg1-positive puncta per cell from three independent experiments. Significant differences with the WT at each time point are indicated. *F*, WT (HAY228), *vps35Δ* (HAY230), *vps1Δ* (HAY304), *atg1Δ* (HAY266), *vps35Δ atg1Δ* (HAY267), and *vps1Δ atg1Δ* (HAY314) strains carrying endogenous *ATG9-GFP* were grown in YPD and imaged as in panel (*A*). *G*, quantification of the experiment depicted in panel (*F*) by determining the number of Atg9-positive puncta per cell from three independent experiments. Significant differences with the WT (*i.e.*, *vps35Δ* and *vps1Δ*) or *atg1Δ* (*i.e.*, vps35Δ atg1Δ and *vps1Δ atg1Δ*) are indicated. All graphs present mean values ± SD. BL, brightfield; DIC, differential interference contrast; mChe, mCherry; n.s., not significant. ∗∗∗*p* < 0.0001. Size bars represent 3 μm.

We first examined the distribution of Atg8, a protein that is recruited to the PAS through conjugation to phosphatidylethanolamine and in part delivered into the vacuole lumen (41). Indeed, mCherry-Atg8 was mainly in puncta in WT cells after 1 h of autophagy induction and mostly localized in the vacuole lumen after prolonged nitrogen starvation (Fig. 2, *A* and *B*). In contrast, the *vps35Δ* mutant showed more mCherry-Atg8 puncta per cell after 1 h and 3 h of nitrogen starvation. In agreement with the GFP-Atg8 processing and Pho8Δ60 assays, the mCherry-Atg8 signal in the vacuole interior was strongly reduced, confirming a severe autophagy defect of the *vps35Δ* knockout (Fig. 2*A*). Compared to *vps35Δ* cells, the *vps1Δ* mutant displayed an even more severe accumulation of mCherry-Atg8 puncta at early and late time points of nitrogen starvation (Fig. 2, *A* and *B*). Accordingly, Atg8 was found to be more lipidated in *vps1Δ* cells than in the WT or *vps35Δ* strains reinforcing the notion of an accumulation of autophagosomal structures in this mutant background (Fig. 2*C*).

The use of Atg8, however, does not allow to distinguish between PAS/autophagosomal intermediates and complete autophagosomes since this protein is present on both structures (41). Therefore, we examined the distribution of the PAS marker proteins Atg1-GFP and Atg16-GFP in *vps35Δ* and *vps1Δ* cells but saw no difference in the number of puncta per cell compared to the WT (Figs. 2, *D* and *E* and S1, *B* and *C*), indicating no defect in the recruitment of these Atg proteins to the PAS.

**Figure 3:**
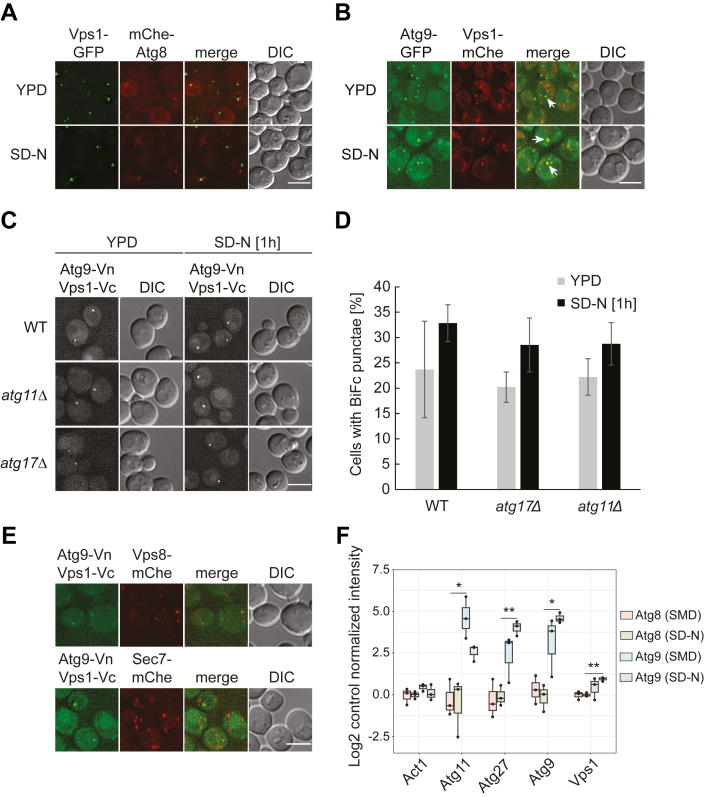
**Atg9 interacts with Vps1.***A*, cells expressing both endogenous Vps1-FL1-GFP and mCherry-V5-Atg8 (BRY260) were grown in YPD and imaged directly or after being incubated in SD-N medium for 1 h. Representative single z-focal planes are shown. *B*, the strain expressing endogenous Atg9-GFP and Vps1-RL2-mCherry-V5 (BRY279) was treated and imaged as in panel (*A*). *Arrows* highlight colocalizations. *C*, WT (BRY304), *atg11Δ* (BRY293), or *atg17Δ* (BRY291) cells coexpressing endogenous Atg9-Vn and Vps1-RL2-Vc were treated and imaged as in panel (*A*). BiFc signals were captured in the YFP channel. *D*, quantification of the experiment shown in panel (*C*) showing the average number of BiFc puncta per cell from three independent experiments. Bars indicate mean values ± SD. *E*, strains expressing Atg9-Vn and Vps1-RL2-Vc with either endogenous Sec7-mCherry-V5 (BRY284) or endogenous Vps8-mCherry-V5 (BRY305) were grown in YPD medium and then starved in SD-N medium for 1 h before imaging. *F*, abundances of the indicated proteins upon APEX2-based Atg9 and Atg8 proximity labeling under rich (SMD without biotin) and starvation (SD-N) conditions. Log2 values of protein intensities are represented. Each protein intensity was normalized to its respective negative control, *i.e.*, the sample without biotinylation reaction. n = 3 biological replicates. ∗: q < 0.05; ∗∗: q < 0.01. Size bars represent 3 μm.

Next, we turned to Atg9, a transmembrane protein involved in delivering the initial membranes of the PAS from the Atg9 reservoirs/Atg9 vesicles (3, 42). In agreement with the literature, Atg9-GFP–expressing WT cells displayed an average of 7 to 9 puncta per cell, one very likely representing the PAS and the other the Atg9 reservoirs (Fig. 2, *F* and *G*; (3, 42)). The number of Atg9-GFP puncta was unaltered in the *vps35Δ* mutant. In the *vps1Δ* knockout, in contrast, their number increased. This specific change in the Atg9 subcellular distribution upon *VPS1* deletion led us to conclude that Vps1 and the retromer are likely involved in two different steps of autophagy.

To test Atg9 trafficking directly, we used the transport of Atg9 after knocking out *ATG1* (TAKA) assay, an epistasis experiment to determine whether a protein acts upstream of the Atg1 kinase complex (43). In an *atg1Δ* strain, recycling of Atg9 from the PAS is blocked, which results in an accumulation of this protein at this structure (44). In contrast, mutants with defects in Atg9 delivery to the PAS do not show this accumulation upon deletion of *ATG1*. The number of Atg9 puncta per cell was strongly reduced in an *atg1Δ* background compared with the WT, reflecting as expected the Atg9 accumulation at the PAS (Fig. 2, *F* and *G*). The number of Atg9 puncta in the *vps35*Δ *atg1Δ* double deletion mutant was comparable to the one measured in *atg1Δ* cells, which indicates that Vps35 acts together or downstream of Atg1. Similar to the *vps1Δ* mutant, the *vps1Δ atg1Δ* mutant showed a high number of Atg9 puncta compared to *atg1Δ* cells (Fig. 2, *F* and *G*). This indicates that Vps1 participates in Atg9 transport to the PAS, upstream of Atg1, and confirms that Vps1 and Vps35 act at separate steps of autophagy. We therefore focused on the interplay between Vps1 and Atg9 in more detail, also because while preparing this manuscript, a recent manuscript showed that Vps35, together with Vps26 and Vps29, is part of a novel retromer complex involved in Atg9 trafficking (45).

### Vps1 interacts with Atg9 at the Atg9 reservoirs

Dynamins are involved in many cellular processes, including different trafficking pathways like endocytosis and exocytosis. Consequently, the observed accumulation of Atg9 in numerous puncta in the *vps1Δ* mutant could also be indirectly caused by impairment in other transport routes. To test for a direct functional connection between Vps1 and Atg9, we first examined whether Vps1 localizes to autophagosomal and/or Atg9-positive membranes by fluorescence microscopy.

Direct fusion of endogenous Vps1 with a tag renders this protein unfunctional and this problem can be solved by inserting an amino acid linker in between (46). We introduced different amino acid linkers, two flexible (*i.e.*, (GGGGS)_3_ and GSAGSAAGSGEF) and two rigid (*i.e.*, A(EAAAK)_6_A and (AP)_33_) (47), and tested the functionality of the resulting fusion proteins by Pho8Δ60 assay (Fig. S2*A* and not shown). The best functional fusion protein per each tag were Vps1-FL1-GFP, Vps1-RL2-mCherry, Vps1-RL2-3xHA, and Vps1-RL2-Vc (Fig. S2*A*).

In fluorescence microscopy, Vps1-FL1-GFP exhibits two distribution patterns. In the one hand, this fusion protein localized to small, rapidly moving vesicles that appear as a blur in images with exposure times higher than 100 ms. On the other hand, it was present in numerous vesicular structures that appear as bright puncta and were less motile. When Vps1-FL1-GFP was coexpressed with mCherry-Atg8, we could detect few puncta that showed overlap between the two fluorescent signals (Fig. 3*A*). We also detected minimal colocalization between endogenous Vps1-RL2-mCherry and Atg9-GFP, indicating that Vps1 is present on either Atg9 reservoirs or PAS/autophagosomal membranes (Fig. 3*B*, arrows).

Based on our observations, we hypothesized that Atg9 could interact with Vps1 to recruit this dynamin onto the Atg9 reservoirs. To test whether these two proteins are adjacent, we first used the split-Venus assay in which two putative interactors are fused with the N-terminal (Vn) and the C-terminal (Vc) fragments, respectively, and a reformation of the Venus signal indicate proximity between the two tagged proteins (48). In control cells expressing either Atg9 or Vps1 with C-terminal tagged Vn or RL2-Vc, no fluorescence signal was detected (Fig. S2*B*). Importantly, Venus-positive puncta were detected in a strain simultaneously expressing Atg9-Vn and Vps1-RL12-Vc in both growth and starvation conditions (Fig. 3, *C* and *D*). We concluded that Vps1 is in close proximity of Atg9. Importantly, this signal was also detected in the *atg11Δ* and *atg17Δ* knockouts, which block formation of the PAS in nutrient-rich and starvation conditions, respectively (11), indicating that Atg9 and Vps1 associate at the Atg9 reservoirs (Fig. 3, *C* and *D*). We also analyzed the Atg9-Vps1-Venus signal in cells expressing marker proteins for endosomes (Vps8-mChe; (49)) or late Golgi (Sec7-mChe; (50)). However, we detected almost no overlap between the Venus signal and the two marker proteins (Fig. 3*E*). This result infers that the Vps1–Atg9 interactions indeed occurs at the Atg9 reservoirs, which are known to not colocalize with various endosomal and Golgi marker proteins (2). To reinforce this notion, we carried out a membrane subcellular fractionation on a sucrose gradient that allows to separate Atg9-positive membranes from the rest of the organelles (2). As shown in Fig. S3, Vps1-RL2-3xHA was also detected in the low-density fractions containing Atg9-13xMYC, reinforcing the notion that Vps1 is present in the Atg9 reservoirs. However, it cannot be excluded that these two proteins could be together in other compartments as well.

Next, we determined whether Vps1 and Atg9 are in close physical proximity by performing an APEX2-based proximity-dependent biotin labeling experiment (51). We also opted for this approach since their interaction could be transient due to the dynamic trafficking of Atg9. Thus, a strain expressing Atg9-APEX2 was either grown in SMD medium lacking biotin or nitrogen starved in SD-N medium for 1 h, before performing the biotinylation reactions as described in Experimental procedures. The APEX2-Atg8–expressing strain (Fig. 3*F*) or the Atg9-APEX2 strain without biotinylation reaction (Fig. S4) were used as negative controls. Cells were subsequently lysed and biotinylated proteins isolated with streptavidin-conjugated beads. Isolated proteins were finally identified by protein mass spectrometry (Figs. 3*F* and S4). Vps1 was identified among known Atg9 binding partners such as Atg9 itself, Atg11, and Atg27 (10, 52, 53).

Altogether, these experiments reveal that Vps1 transiently associates directly or indirectly with Atg9 at the Atg9 reservoirs, and it is involved in the transport of this transmembrane protein to the PAS.

### Vps1 is required for autophagosome formation

**Figure 4:**
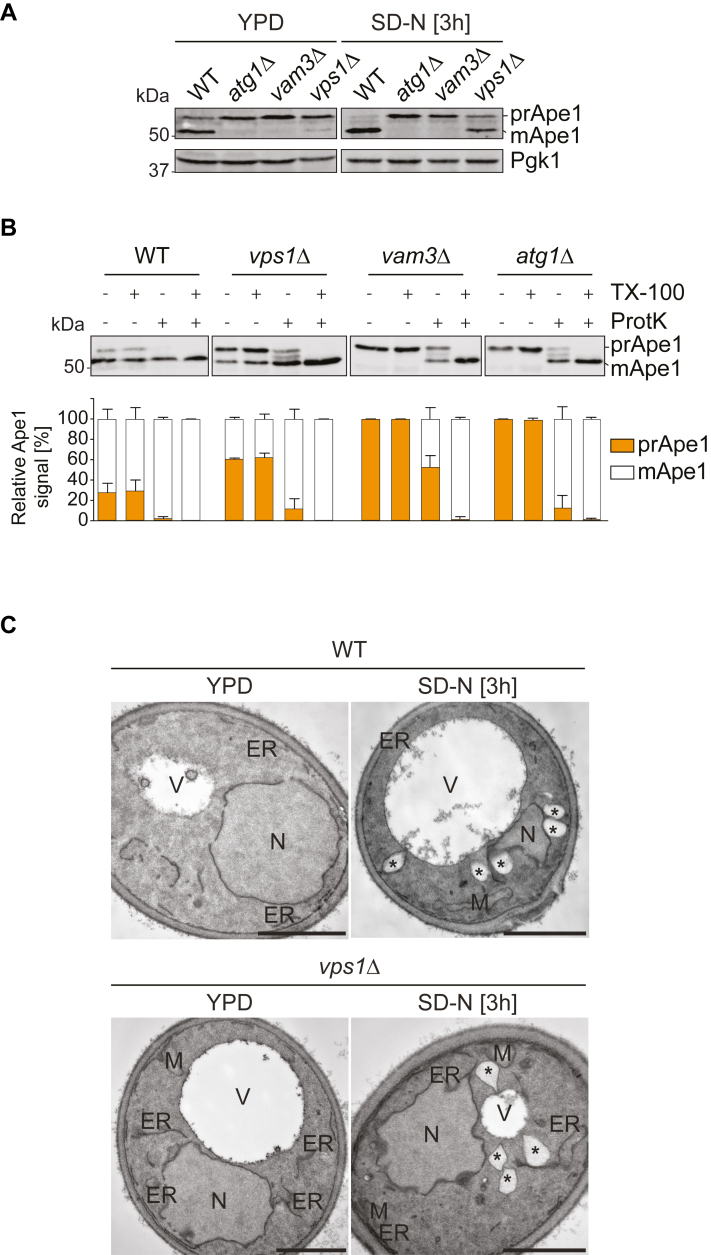
**Vps1 function is required for autophagosome formation.***A*, the WT (SEY6210), *atg1Δ* (WHY1), *vam3Δ* (CWY10), and *vps1Δ* (HAY294) strains were grown in YPD before transfer to SD-N for 3 h to induce autophagy by nitrogen starvation. Cells were collected before and after nitrogen starvation, and proteins were analyzed by Western blot using anti-Ape1 and anti-Pgk1 antibodies. Pgk1 served as the loading control. *B*, the Ape1 protease protection assay was carried out with the WT (SEY6210), *atg1Δ* (WHY1), *vam3Δ* (CWY10), and *vps1Δ* (HAY294) strains starved for nitrogen as described in Experimental procedures. Quantification of the relative amount of prApe1 and mApe1 in three independent experiments ± SD is shown below the representative blot. *C*, WT (SEY6210) and *vps1Δ* (HAY294) cells grown in YPD or starved in SD-N medium for 3 h were examined at the ultrastructural level by electron microscopy. *Asterisks* indicate lipid droplets. Size bar represents 1 μm. TX-100, Triton X-100; ProtK, proteinase K; ER, endoplasmic reticulum; M, mitochondria; N, nucleus; V, vacuole.

To determine more precisely the step of autophagy involving Vps1, we first examined whether deletion of this protein impacts the Cvt pathway, a biosynthetic type of selective autophagy, which also relies on the core Atg machinery (54). The major cargo molecule of the Cvt pathway is the vacuolar peptidase Ape1, which is synthesized as a 61 kDa prApe1 precursor and upon delivery into the vacuole, is processed into its 50 kDa Ape1 mature form (54). This change in Ape1 size allows assessing the functionality of the Cvt pathway by Western blot. Thus, we analyzed Ape1 transport to the vacuole in the *vps1Δ* mutant. As a control, we used *atg1Δ* and *vam3Δ* cells, in which prApe1 maturation is blocked because of a defect in autophagosome formation and autophagosome fusion with the vacuole, respectively (16, 55, 56, 57). As expected, prApe1 processing was almost complete in WT cells and blocked in the *atg1Δ* and *vam3Δ* strains in both rich medium and nitrogen starvation conditions (Fig. 4*A*). Interestingly, the *vps1Δ* knockout showed an impairment in prApe1 processing in both conditions, albeit more exacerbated in the presence of nutrients. This shows that Vps1 also plays an important role in the Cvt pathway, suggesting a function of this protein in selective types of autophagy as well.

Next, we asked whether the prApe1 that accumulated in the *vps1Δ* mutant is cytoplasmic, that is, autophagosomes cannot form properly or whether it is in the interior of complete autophagosomes, that is, these vesicles can form but they are unable to fuse with the vacuole. For this, we performed a protease protection assay, which determines whether prApe1 is enwrapped into an autophagosome and therefore is not accessible to exogenous proteases (58). To distinguish these two situations, we once again took advantage of the *atg1Δ* and *vam3Δ* mutants. WT cells mostly accumulated the mApe1, with little prApe1 even in the absence of exogenous Proteinase K, which proves that prApe1 is normally targeted *via* Cvt pathway to the vacuole where it is processed. All the prApe1 was accessible to protease treatment event without detergent (Fig. 4*B*). When the cell extract from *vam3Δ* cells was incubated with proteinase K, approximately 50% of prApe1 was protected and processed only upon addition of detergent, showing, as expected, that part of the prApe1 is protected inside autophagosomes (Fig. 4*B*). In contrast, prApe1 was almost completely processed in cell extracts from the *atg1Δ* knockout in the presence of Proteinase K, in which accumulated prApe1 is not sequestered and therefore protected inside an autophagosome (Fig. 4*B*). Importantly, prApe1 was accessible to proteinase K in cell extracts from *vps1Δ* cells to a similar extent as observed for those obtained from the *atg1Δ* knockout (Fig. 4*B*). This result indicates that cells lacking Vps1 are unable to generate complete autophagosomes.

To substantiate this finding, we inspected the ultrastructure of the *vps1Δ* mutant in logarithmic growth and upon nitrogen starvation by electron microscopy, to look for accumulation of phagophores or aberrant autophagosomal intermediates, but also to exclude that *VPS1* deletion affects autophagy by impairing autophagosome fusion with the vacuole. The profiles of the *vps1Δ* mutant displayed no difference with those of the WT cells (Fig. 4*C*). In particular, no accumulation of autophagosomes or eventually expanded phagophores was observed (Fig. 4*C*).

Altogether, these data show that Vps1 function is required for the early steps of autophagosome formation, which agrees with the notion that Vps1 is required for Atg9 transport to the PAS.

### Vps1 function- and disease-associated point mutants severely impair autophagy

**Figure 5:**
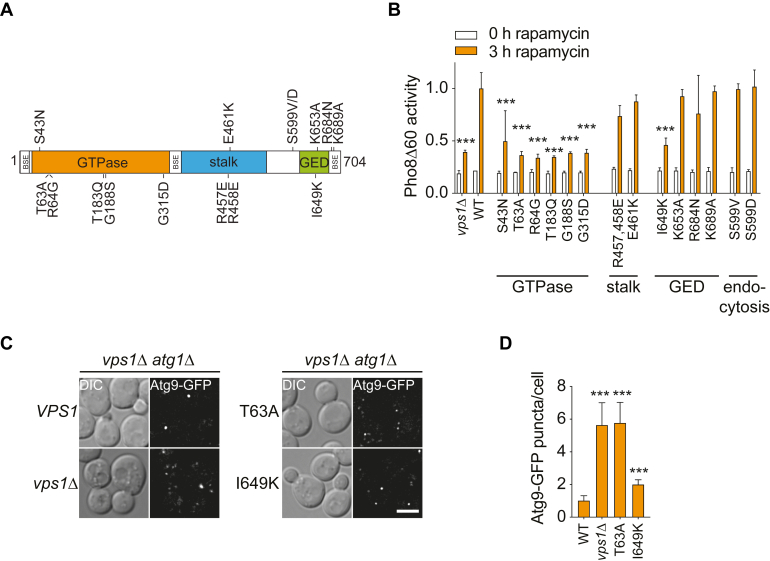
**Vps1 GTPase and oligomerization mutants block autophagy.***A*, overview of the structural organization of Vps1. Functional mutations examined in this study are indicated. *B*, the Pho8Δ60 assay was carried in the *vps1Δ* mutant (HAY164) transfected with either an empty vector (vps1Δ) or plasmids carrying VPS1 mutants (Table 1), as in Figure 1*G*, although cells were grown in SMD medium lacking uracil. Data represent the average of three independent experiments expressed relatively to the WT treated with rapamycin for 3 h. The graphs indicate mean values ± SD, and significant differences of the rapamycin-treated samples with the WT are indicated. ∗∗∗*p* < 0.0001. *C*, the *vps1Δ atg1Δ* (HAY314) strain expressing endogenous ATG9-GFP was transformed with an empty vector (*vps1Δ*) or plasmids expressing Vps1, Vps1(T63A), and Vps1(I649K) was grown in SMD medium lacking uracil and imaged as in Figure 2*F*. Representative single plains of GFP-fluorescence z-stacks are shown. Size bar represents 3 μm. *D*, quantification of the Atg9-GFP puncta per cell in the experiment shown in panel (*C*) from three independent experiments. Significant differences with the WT are indicated. All graphs present mean values ± SD. DIC, differential interference contrast. ∗∗∗*p* < 0.0001.

Vps1, and more generally dynamins, have a N-terminal GTPase domain, three helix bundle (BSE) domains, and a middle, stalk domain formed by helix bundles and GTPase effector domain (Fig. 5*A*) (59). To gain further insight into how Vps1 functionally participates in autophagy, we expressed several previously characterized Vps1 point mutants that affect both yeast Vps1 and higher eukaryotic dynamins (Table 1 and Figs. 5*A* and S5; (60)). We also included two point mutants, that is, S599V, S599D, known to cause an endocytosis defect in yeast without affecting other trafficking pathways (61). We expressed the different Vps1 mutants from a plasmid in *vps1Δ* cells and assessed autophagic flux using the Pho8Δ60 assay. Single point mutations in the GTPase domain (S43N, T63A, R64G, T183Q, G188S, and G315D) were not able to rescue the Pho8Δ60 defect observed in knockout cells, while mutations in the middle stalk domain (R457E, R458E, and E461K) had no effect on autophagy (Fig. 5*B*). We also tested several mutants in the GTPase effector domain that are known to affect self-assembly (I649K, K653A, R684N, K689A) (62, 63). Interestingly, only the I649K, which causes the strongest defect in higher eukaryotes (62), was unable to rescue the autophagy defect of the *vps1Δ* mutant (Fig. 5*B*).

**Table 1:** Overview of function-associated Vps1 mutations examined in this study

Yeast mutation	Domain	Human mutation	Information	Reference
S43N	GTPase	DNM1(S45N)	GTPase activity	(63, 95)
T63A	GTPase	DNM1(T65A)	Low GTP affinity mutation in DNM1	(95, 96)
R64G	GTPase	DNM1(R66A)	GTPase activity	(63, 95)
T183Q	GTPase	DNM1(T141Q)	Partial GTPase defect	(63, 95)
G188S	GTPase	DNM1 (G146S)	GTP-binding; *shibire ts2*	(63, 97)
G315D	GTPase	DNM1 (G273D)	*shibire ts1*	(63, 98)
R457,458E	middle		Endocytic vesicle scission defect	(46)
E461K	middle		Reduced endocytosis	(84)
S599V	-	Endocytosis defect	(61)	
S599D	-	Endocytosis defect	(61)	
I649K	GED	DNM1(I690K)	Self-assembly defect, endocytosis defect in yeast	(62, 63, 85)
K653A	GED	DNM1(K694A)	Impaired self-assembly	(63)
R684N	BSE	DNM1(R725A)		(20, 99)
K689A	BSE	DNM1(K730A)		(20, 99)

We asked whether the autophagy defect of the GTPase- and oligomerization-deficient variants was due to an impairment of the Atg9 transport to the PAS. To test this, we expressed the GTPase-defective T63A and oligomerization-deficient I649K mutants in a *vps1Δ atg1Δ* strain and performed the TAKA assay. While the I649K mutant displayed a mild, albeit significant increase in Atg9-positive structures, that is, a partial block in Atg9 delivery to the PAS, the T63A GTPase mutant showed a strong increase comparable to the empty vector control (Fig. 5, *C* and *D*). We observed similar defects of these mutants when the same experiment was performed under nitrogen starvation conditions (Fig. S5). Therefore, we concluded that Vps1 GTPase activity and oligomerization are required for Atg9 transport to the PAS and autophagy.

**Figure 6:**
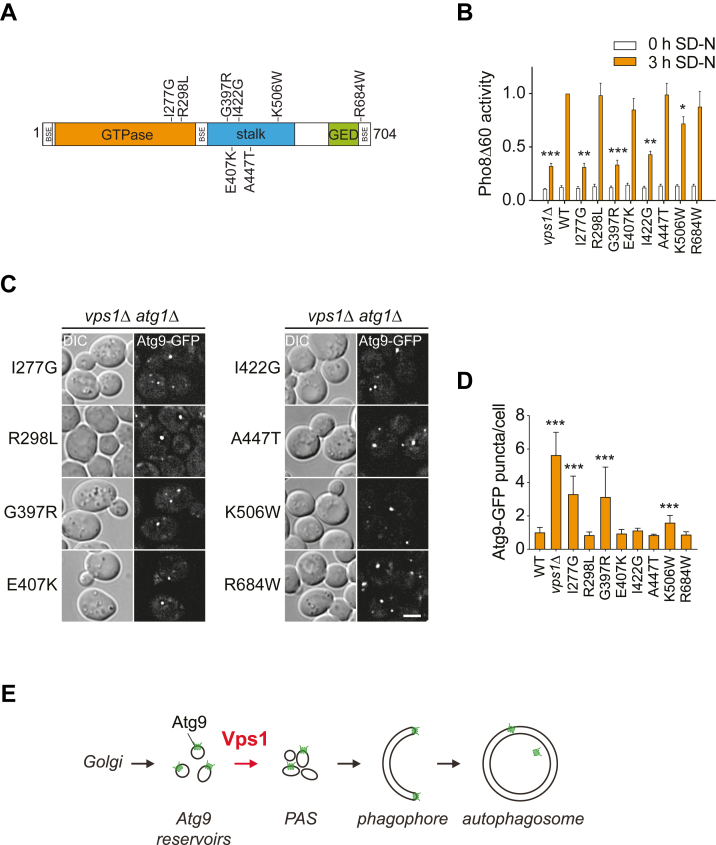
**Specific Vps1 variants associated with human diseases block autophagy.***A*, overview of the structural organization of Vps1. Disease-associated mutations examined in this study are indicated. *B*, the Pho8Δ60 assay was carried out in *vps1Δ* mutant (HAY164) transfected with either empty vector (pRS416) or plasmids carrying *VPS1* mutants (Table 2), as in Figure 1*G*, although cells were grown in SMD medium lacking uracil. Data represent the average of three independent experiments expressed relatively to the WT treated with rapamycin for 3 h. Graphs indicate mean values ± SD, and significant differences of the rapamycin-treated samples with the WT are indicated. ∗*p* < 0.015; ∗∗*p* < 0.0015; ∗∗∗*p* < 0.0001. *C*, the *vps1Δ atg1Δ* (HAY314) strain carrying endogenous *ATG9-GFP* and transformed with an empty vector (pRS416) or plasmids expressing the indicated Vps1 variants was grown in SMD medium lacking uracil and imaged as in Figure 2*F*. Representative single image planes of GFP-fluorescence z-stacks are shown. *D*, quantification of the Atg9-GFP puncta per cell in the experiment shown in panel (*C*) from three independent experiments. Significant differences with the WT are indicated. ∗∗∗*p* < 0.0001. All graphs present mean values ± SD. DIC, differential interference contrast. Size bar represents 3 μm.

Mammalian cells have three dynamin isoforms, DNM1, DNM2, and DYNAMIN3 (DNM3), with a variety of expression patterns and proposed functions (19). Mutations identified in these proteins are known to cause specific neurological disorders and other pathologies in humans ((20, 64), Table 2). While defects in endocytosis and endomembrane trafficking have been implicated in the etiology of these diseases, we wondered if some of these mutations might cause a defect in autophagy as well. Due to the high conservation between human dynamins and yeast Vps1, with approximately 32% amino acid sequence identity, we identified all the corresponding mutations reported to cause the Charcot-Marie-Tooth disorder, epileptic encephalopathy, microcytic anemia, centronuclear myopathy, hereditary spastic paraplegia, and lethal congenital contracture syndrome, but also exercise-induced collapse in Labradors and epilepsy in the fitful mouse model in Vps1 (Fig. S6 and Table 2). We then expressed Vps1 variants for several of these pathologies, including microcytic anemia (I277G), Charcot-Marie-Tooth disorder (G397R), centronuclear myopathy (E407K, I422G, and K506W), hereditary spastic paraplegia (R684W), exercise-induced collapse in Labradors (R298L), and a candidate mutation for epilepsy in humans (A447T) in *vps1Δ* cells to examine autophagic flux using again the Pho8Δ60 assay (Fig. 6*A* and Table 2). Interestingly, three Vps1 mutants, I277G, G397R, and I442G, corresponding to DNM1 V235G, DNM2 G358R, and DNM2 V375G mutations found in microcytic anemia, Charcot-Marie-Tooth, and centronuclear myopathy, respectively, showed a defect in autophagic flux (Fig. 6*B*).

**Table 2:** Overview of the disease-associated Vps1 mutations examined in this study

Yeast mutation	Domain	Human mutation	Information	Reference
n.c.	GTPase	DNM1(A117P)	Epileptic encephalopathy	-
n.c.	GTPase	DNM1(K206N)	Epileptic encephalopathy	-
I277G	GTPase	DNM2(V235G)	Microcytic anemia	This study
R298L	GTPase	DNM1(R256L)	Exercise-induced collapse in Labradors	(60)
G397R	middle	DNM2(G358R)	Charcot-Marie-Tooth disorder	(60)
A447T	middle	DNM1(A408T)	Epilepsy in fitful mouse model	(60)
n.c.	middle	DNM2(F379V)	Lethal congenital contracture syndrome	-
n.c.	middle	DNM1(G359A/R)	Epileptic encephalopathy	-
n.c.	middle	DNM2(G359D)	Charcot-Marie-Tooth disorder	-
E407K	middle	DNM2(E368K/Q)	Centronuclear myopathy	This study
n.c.	middle	DNM2(R369Q/W)	Centronuclear myopathy	-
I422G	middle	DNM2(V375G)	Centronuclear myopathy	This study
K506W	middle	DNM2(R465W)	Centronuclear myopathy	This study (20),
n.c.	GED	DNM2(P647R)	Centronuclear myopathy	-
n.c.	GED	DNM2(E650K)	Centronuclear myopathy	-
R684W	GED	DNM2(R719W)	Hereditary spastic paraplegia	This study

n.c., not conserved.

Finally, we tested whether the disease-associated variants impair Atg9 transport to the PAS. For this, we turned once more to the TAKA assay and expressed all these variants in the *vps1Δ atg1Δ* cells carrying endogenous Atg9-GFP. All Vps1 point mutants without a Pho8Δ60 processing defect displayed normal Atg9-GFP delivery to the PAS (Fig. 6, *C* and *D*). In contrast, the I277G and G397R Vps1 variants showed an accumulation of Atg9-positive puncta, indicative of an Atg9 transport defect to the PAS (Fig. 6, *C* and *D*). This result confirms that Vps1-mediated Atg9 trafficking to the PAS is crucial for autophagy progression. Interestingly, like the I649K mutant that displays a severe autophagic flux defect (Fig. 5, *B*–*D*), the I442G Vps1 variant did not impair Atg9 transport to the PAS (Fig. 6, *C* and *D*), suggesting that Vps1 may also participate to other steps of autophagy.

Next, we examined whether the I277G and G397R mutations that impair Atg9 transport affect other cellular functions of Vps1. While G397R and I277G variants complemented the vacuole fission defect of *vps1Δ* cells (65), they could not bypass their vacuolar protease sorting impairment (66) (Fig. S7, *A* and *B*). In addition, the G397R Vps1 variant was able to restore normal GFP-Snc1 cycling in the vps1Δ mutant (63), while the I277G mutant could not Fig. S7*C*). These results show that the G397R and I277G mutations do not exclusively alter the Vps1 function in autophagy.

Altogether, our data infers that an autophagy defect may contribute to the pathophysiology of specific human diseases caused by mutation in dynamins.

## Discussion

In this study, we unraveled a novel link between Atg9 and Vps1 in autophagy. By screening all *SNX* mutants in yeast for autophagic flux, we identify defects in several retromer mutants and subsequently Vps1 (Fig. 1). This could be explained by the fact that Vps1 cooperates with the retromer complex in specific recycling transport steps within the endocytic pathway (37, 38). However, our phenotypic analysis indicates that Vps1 and retromer act along different pathways in autophagy. In particular, our data revealed that *vps1Δ* mutant affects transport of Atg9 vesicles, leading defects in autophagosome biogenesis (Figs. 2 and 4). We demonstrate that Vps1 colocalizes and is in close proximity of Atg9 (Fig. 3) and that Vps1 GTPase activity is required to rescue the autophagy defect of *vps1Δ* cells (Fig. 5). Furthermore, we provide indication that specific DNM1 and DNM2 mutations, known to cause human diseases, may be connected with an autophagy defect, since *VPS1* variants carrying homolog mutations severely impair autophagic flux and lead to a defect in Atg9 transport to the PAS and autophagic flux impairment (Fig. 6).

Vps1 is known to catalyze membrane fission reactions (19), but it has also been implicated in the regulation of fusion by binding to the SNARE Vam3 (65, 67). In mammalian cells, ATG9A trafficking from recycling endosomes during autophagy requires SNX18 and DNM2, but also the BAR domain–containing BIF1 (31, 32, 33, 68). Both SNX18 and BIF1 recruit DNM2 onto recycling endosome membranes (32, 33), but it remains unclear if they are working cooperatively. Another SNX complex, consisting of SNX4-SNX7, was also shown to release ATG9A from endosomes (69). Interestingly, we also observe an autophagy defect in the SNX mutants *vps5Δ* and *vps17Δ* (Fig. 1). However, these cells display a different autophagy defect than the *vps1Δ* mutant (Figs. 1 and 2). Thus, it is unlikely that Vps1 cooperates with the retromer in the transport of Atg9 from its reservoirs to the PAS, but we cannot exclude that these two components could act together at another step of autophagy. In this regard, it has recently been revealed that Vps26, Vps29, and Vps35, together with Atg18 but not Vps5 and Vps17, are part of a novel retromer complex (45, 70). Thus, one could imagine that Vps1 cooperate with this complex as it does with the well-established retromer (37, 38).

Our screen did not identify other yeast SNX involved in autophagy, and redundancy does not appear to play a role, since double mutants, combining SNX deletions, did not show an autophagy defect. Other studies in yeast have concluded that SNX, and in particular the retromer, are not involved in autophagy unless they are codepleted with other redundant endosomal recycling pathways (25, 71). The retromer is required to retrieve Atg9 from endosomes to the Golgi (25, 45, 71), consistent with what has been also reported for mammalian ATG9A (23). This apparent discrepancy with our data is very likely because the two previous studies have assessed the autophagic flux only by looking at prApe1 maturation instead of bulk autophagy (35). prApe1 is a cargo specifically targeted to autophagosomes and hence, it is also delivered into the vacuole in mutants that display a severe autophagy impairment but not a complete block (54). Examples of this type of mutants include *vac8Δ* cells (72). One of the two recent studies on the novel retromer complex, however, found that deletion of each one of the subunits of the retromer severely affects bulk autophagy (45), in complete agreement with our data. As Atg9 levels and possibly its trafficking directly correlates with the frequency of autophagosome formation (73), we favor the hypothesis that the severe impairment of Atg9 transport to the PAS in *vps1Δ* cells strongly reduces the frequency of autophagosome formation. Nevertheless, additional studies are needed to determine which sorting factors, other than SNX, are involved in the Vps1-mediated Atg9 trafficking step.

How does Vps1 act during autophagy? The phenotypes of the *VPS1* mutants cannot be simply explained by an impairment in autophagosome-vacuole fusion (Fig. 4) nor by a defect in fission during Atg9 vesicle biogenesis (Fig. 2). Vps1 acts in several different cellular processes, which makes it difficult to delineate the phenotype of the corresponding knockout to a specific pathway. Consistently with what is found in mammalian cells (32, 33, 74), our data support the notion that Vps1 acts directly in autophagy since we observed both a specific defect in Atg9 reservoir transport to the PAS in the absence of this dynamin (Fig. 2, *F* and *G*) and Vps1 localization to the Atg9 reservoirs (Fig. 3, *B*–*D*). Moreover, we show here that Vps1 interacts directly or indirectly with Atg9 (Fig. 3, *C*, *D* and *F*). In mammalian cells, DNM2 is recruited to ATG9A-containing membranes *via* binding to SNX18, BIF1, and/or the autophagic adapter LC3 (32, 33, 74). Future investigations need to address how the Vps1-Atg9 association in yeast is regulated and coupled to Vps1 function in autophagy. For this, it will be important to identify the domain in Vps1 and/or Atg9 involved in their interaction and generate specific binding point mutants. Since the Vps1–Atg9 interaction may be not direct, identification of the possible bridging protein(s) will provide insights into the role of Vps1 function in autophagy.

Several mutations causing neurobiological diseases have been directly or indirectly associated with a defect in autophagy, which leads to an accumulation of unwanted cytoplasmic structures, including cell-toxic aggregates and damaged organelles (75, 76, 77). While this manuscript was in preparation, a new study revealed that the R465W mutation in DNM2, which causes centronuclear myopathy, has been associated to an autophagy defect characterized by an impairment in autophagosome formation (74). The homologous mutation in yeast, that is, K506W (Fig. S6 and Table 2), leads only to a weak defect in Atg9 trafficking. However, one of the three variants that we identified as defective in autophagy, that is, G397R, which corresponds to the DNM2(G358R) mutation causing Charcot-Marie-Tooth disorder (60, 78) (Fig. S6 and Table 2), is also localized in the stalk region of Vps1 and DNM2. Consistently, we also found that the G397R change in Vps1 leads to a defect in autophagosome formation. The second Vps1 variant that we found to affect autophagy progression is I277G, which mimics the DNM2(V235G) mutation causing microcytic anemia (21) (Fig. S6 and Table 2). The I277G lies in the GTPase domain of Vps1 and consequently, like the V235G change (21), it probably impairs the GTPase activity of this protein. This agrees with our finding that the GTPase domain of Vps1 is essential for its role in autophagy (Fig. 5*B*) but also the fact that the I277G affect the endosomal functions of Vps1 (Fig. S7). Most of the mutations affecting dynamin oligomerization appear to not interfere with autophagy flux (Fig. 5*B*), and this observation is somewhat surprising since dynamin oligomerization is essential for most of the functions of this protein family, including endocytosis (63). However, the mutation known to affect dynamin oligomerization the most (62, 63), that is, I649K, severely inhibits autophagy without having a major impact on Atg9 trafficking (Fig. 5, *B*–*D*). This result suggests that Vps1 could also participate in a step of autophagy epistatic to the Atg9 transport to the PAS. This notion is also sustained by our identification of a mutation in Vps1 stalk region, I442G, corresponding to the human variant DNM2(V375G) causing centronuclear myopathy (Table 2), which displays a severe defect in autophagic flux but normal Atg9 transport to the PAS (Fig. 6, *B*, *C* and *D*).

Overall, our data complement and extent those obtained in mammalian cells in which Vps1, as DNM2, is required for Atg9 trafficking (32, 33). We additionally show that it is essential for Atg9 to be delivered to the site of autophagosome formation. In this context, it is interesting to note that the disease-associated mutants that we found to be defective in Atg9 transport have been reported to be in DNM2 (21, 60, 78). Thus, our study supports the recent finding that a defect of autophagy probably plays a role in the pathophysiology of diseases caused by mutations in DNM2 and suggest that those could include microcytic anemia and Charcot-Marie-Tooth diseases, in addition to centronuclear myopathy.

## Experimental procedures

### Yeast strains, media, and plasmids

Genetic manipulation in yeast *S. cerevisiae* was performed by homologous integration of PCR-amplified DNA cassettes (48, 79, 80). Gene knockouts or taggings were verified by Western blot using specific antibodies and/or PCR analysis of the modified gene locus. Yeast strains used in this study are listed in Table 3. Yeast cells were grown in rich (YPD; 1% yeast extract, 2% peptone, and 2% glucose) or synthetic minimal (SMD; 0.67% yeast nitrogen base, 2% glucose, and amino acids and vitamins as needed) medium. Autophagy was induced by transferring cells into a nitrogen starvation medium (SD-N; 0.17% yeast nitrogen base without amino acids and ammonium sulfate and 2% glucose) or by addition of 0.2 μg/ml rapamycin (Sigma).

**Table 3:** Yeast *Saccharomyces cerevisiae* strains used in this study

Strain	Genotype	Source
BRY239	WLY176 *VPS1-FL1-GFP*::*HIS3*	This study
BRY260	SEY6210 *VPS1-FL1-GFP*::*HIS3 pmCheV5ATG8406*::*URA3*	This study
BRY273	YTS159 *VPS1-RL2-mCherryV5*::*LEU2*	This study
BRY277	YTS159 *VPS1-RL2-3xHA::LEU2*	This study
BRY279	SEY6210 *ATG9-GFP*::*TRP1 VPS1-RL2-mCherryV5*::*LEU2*	This study
BRY283	SEY6210 *ATG9-13xMYC::HIS3 VPS1-RL2-3xHA*::*LEU2*	This study
BRY284	SEY6210 *ATG9-VN*::*HIS3 VPS1-RL2-VC*::*LEU2 SEC7-mCherryV5*::*TRP1*	This study
BRY291	SEY6210 *ATG9-VN*::*HIS3 VPS1-RL2-VC*::*LEU2 atg17Δ*::*hphMX6*	This study
BRY293	SEY6210 *ATG9-VN*::*HIS3 VPS1-RL2-VC*::*LEU2 atg11Δ*::*hphMX6*	This study
BRY299	YTS159 *VPS1-RL2-VC*::*LEU2*	This study
BRY304	SEY6210 *ATG9-VN*::*TRP1 VPS1-RL2-VC*::*LEU2*	This study
BRY305	SEY6210 *ATG9-VN*::*HIS3 VPS1-RL2-VC*::*LEU2 VPS8-mCherryV5*::*TRP1*	This study
BRY306	SEY6210 *VPS1-RL2-VC*::*LEU2*	This study
BY4741	*MATa his3Δ1 leu2Δ0 met15Δ0 ura3Δ0*	Euroscarf
BY4742	*MATα his3Δ1 leu2Δ0 lys2Δ0 ura3Δ0*	Euroscarf
BY4727	*MATα his3Δ200 leu2Δ0 lys2Δ0 met15Δ0 trp1Δ63 ura3Δ0*	Euroscarf
CWY40	SEY6210 *vam3Δ*::*TRP1*	(100)
FRY244	*MATa his3Δ1 leu2Δ0 lys2Δ0 ura3Δ0 pho13Δ*::*kanMX pho8*::*PHO8Δ60 atg9Δ*::*URA3S.p.LoxP*	(2)
HAY8	BY4742 *vps1Δ*::*kanMX6*	Euroscarf
HAY9	BY4742 *dnm1Δ*::*kanMX6*	Euroscarf
HAY11	BY4742 *atg24Δ*::*kanMX6*	Euroscarf
HAY12	BY4742 *atg20Δ*::*kanMX6*	Euroscarf
HAY26	BY4741 *vps5Δ*::*kanMX6*	Euroscarf
HAY29	BY4741 *atg24Δ*::*kanMX6*	Euroscarf
HAY31	BY4741 *mvp1Δ*::*kanMX6*	Euroscarf
HAY32	BY4741 *ykr078wΔ*::*kanMX6*	Euroscarf
HAY33	BY4741 *snx3Δ*::*kanMX6*	Euroscarf
HAY34	BY4741 *vps17Δ*::*kanMX6*	Euroscarf
HAY35	BY4741 *vps26Δ*::*kanMX6*	Euroscarf
HAY36	BY4741 *vps35Δ*::*kanMX6*	Euroscarf
HAY38	BY4741 *snx41Δ*::*kanMX6*	Euroscarf
HAY39	BY4741 *vps5Δ*::*kanMX6*	Euroscarf
HAY43	BY4741 *vps29Δ*::*kanMX6*	Euroscarf
HAY48	BY4741 *ypr097wΔ*::*kanMX6*	Euroscarf
HAY51	BY4741 *ypt35Δ*::*kanMX6*	Euroscarf
HAY161	YTS159 *vps5Δ*::*hphNT1*	This study
HAY162	YTS159 *vps17Δ*::*hphNT1*	This study
HAY163	YTS159 *vps35Δ*::*hphNT1*	This study
HAY164	YTS159 *vps1Δ*::*hphNT1*	This study
HAY196	SEY6210 *ATG9-VN*::*HIS3*	This study
HAY205	YTS159 *vps29Δ*::*hphNT1*	This study
HAY211	SEY6210 *pmCheV5ATG8406*::*URA3*	This study
HAY230	SEY6210 *ATG9-GFP*::*TRP1 pmCheV5ATG8406*::*URA3 vps35Δ*::*hphNT1*	This study
HAY245	YTS159 *vps26Δ*::*hphNT1*	This study
HAY253	SEY6210 *pmCheV5ATG8406*::*URA3 vps35Δ*::*hphNT1*	This study
HAY254	SEY6210 *vps35Δ*::*hphNT1*	This study
HAY266	SEY6210 *ATG9-GFP*:: *pmCheV5ATG8406*::*URA3 atg1Δ*::*HIS3*	This study
HAY267	SEY6210 *ATG9*::*GFP-TRP1 vps35Δ*::*hphNT1 pHA128*::*URA3 atg1Δ*::*HIS3*	This study
HAY294	SEY6210 *vps1Δ*::*hphNT1*	This study
HAY296	SEY6210 *pmCheV5ATG8406*::*URA3 vps1Δ*::*hphNT1*	This study
HAY299	SEY6210 *pmCheV5ATG8406*::*URA3 VPS1-mGFP*::*kanMX6*	This study
HAY303	SEY6210 *ATG9*::*GFP-TRP1 vps1Δ*::*hphNT1*	This study
HAY304	SEY6210 *ATG9*::*GFP-TRP1 vps1Δ*::*hphNT1 pmCheV5ATG8406*::*URA3*	This study
HAY314	SEY6210 *ATG9*::*GFP-TRP1 vps1Δ*::*hphNT1 atg1Δ*::*HIS3*	This study
RSGY044	SEY6210 *ATG1-GFP*::*TRP1*	This study
RSGY045	SEY6210 *ATG16-GFP*::*TRP1*	This study
RSGY046	SEY6210 *ATG1-GFP*::*TRP1 vps35Δ*::*natNT2*	This study
RSGY047	SEY6210 *ATG1-GFP*::*TRP1 vps1Δ*::*hphNT1*	This study
RSGY048	SEY6210 *ATG16-GFP*::*TRP1 vps35Δ*::*natNT2*	This study
RSGY049	SEY6210 *ATG16-GFP*::*TRP1 vps1Δ*::*hphNT1*	This study
SEY6210	*MATa ura3-52 leu2-3112 his3-Δ200 trp1-Δ901 lys2-801 suc2-Δ9 mel GAL*	(101)
YFMLY141	YSBN5 *hphNT1*::*KanMX6-FLAG-APEX2-NES-FL1-ATG8 atg8Δ*::*hphNT1*	This study
YFMLY170	YSBN5 *ATG9-FLAG-APEX2-NES*::*NatMX6*	This study
YSBN5	*MATa* ho::l*oxP-TEF1p-ble-TEF1t-loxP ura3-52*::*URA3*	Euroscarf
YTS159	*MATa his3Δ1 leu2Δ0 lys2Δ0 ura3Δ0 pho13Δ*::*kanMX pho8*::*PHO8Δ60*	(102)
YTS161	YTS159 *atg1Δ*::*HIS5 S.p.*	(102)
WHY1	SEY6210 *atg1Δ*::*HIS5 S.p.*	(103)
WLY176	SEY6210 *pho13Δ pho8*::*PHO8Δ60*	(104)

The different tags with a linker to generate endogenous C-terminally Vps1 fusion proteins were created as follows. GFP, 3xHA, mCherryV5, and Vc tags were generated by PCR using 5′ primers that inserted the amino acid linker (GGGGS)_4_ (flexible linker 1, FL1), GSAGSAAGSGEF (flexible linker 2, FL2), A(EAAAK)_6_A (rigid linker 1, RL1), or (AP)_33_ (rigid linker 2, RL2) (47), before to be cloned as PacI/AscI fragments into the pFA6A plasmid series that is used as a template for endogenous tagging by PCR-based recombination (80). The FLAG-APEX2-NES tag was cloned into the pFA6A plasmids using the same strategy upon PCR amplification from the pDP287 vector, and the resulting plasmid were then used as a template to generate the strain expressing Atg9-FLAG-APEX2-NES by homologous recombination (80).

Chromosomal tagging of *ATG8* at its 5′ end with the FLAG-APEX2 tag was realized by replacing the *hphNT1 cassette* used to knock out the *ATG8* gene with the *KanMX6-ATG8pr-FLAG-APEX2-NES-FL1-ATG8* by homologous recombination through 1000 bases of identity upstream the *ATG8* promoter and downstream the *ATG8* gene. This module was generated by PCR from the pYFMLP034 plasmid, which was generated by sequentially cloning 1000 bp of the homology region upstream the *ATG8* promoter, the drug resistance gene *NatMX6*, the *ATG8* promoter, the *FLAG-APEX2-NES* tag, *ATG8* gene, and 1000 bp of the homology region downstream the *ATG8* gene into the pRS404 vector.

Plasmids pRS404, pRS416, pCuGFPAUT7(416), pmCheV5ATG8406, and pGS416 have been described elsewhere (2, 81, 82, 83), as well as those expressing WT Vps1 (pKA677) and the following Vps1 point mutants: S43N (pKA692), R684N (pKA693), G188S (pKA697), G315D (pKA698), T63A (pKApKA699), T183Q (pKA700), I649K (pKA701), K653A (pKA702), K689A (pKA703), R64G (pKA707), S599V (pKA781), S599D (pKA782), A447T (pKA796), G397R (pKA797), R298L (pKA798), RR457,458EE (pKA943), and E461K (pKA945) (46, 61, 63, 84, 85), (Tables 1 and 2). The plasmids expressing the I277G (pVPS1(I277G)), E407K (pVPS1(E407K)), I422G (pVPS1(I422G)), K506W (pVPS1(I506W)), and R684W (pVPS1(R684W)) were created by site-directed mutagenesis of the pKA677 vector using appropriate primers.

The centromeric plasmid pVPS1(ts)416 carrying *vps1-100* was created by excising this thermosensitive *VPS1* allele from the pCAV40 vector (40) and insert it into pRS416 using NotI and ClaI.

### Biochemical assays

GFP-Atg8 processing and Pho8Δ60 activity measurements were carried out as previously described (35). Monoclonal anti-GFP antibodies were from Roche. Atg8 lipidation and Ape1, Prc1, and Pep4 maturation and processing were assessed by separating trichloroacetic acid (TCA)-precipitated proteins by SDS-PAGE and after Western blot, probing the membranes with anti-Atg8, anti-Pgk1, anti-Ape1, anti-Prc1, and anti-Pep4 polyclonal antibodies (2, 17, 86, 87). Cells were grown in a medium buffered with 100 mM sodium citrate (pH 4.0) for the examination of Prc1 and Pep4 (88).

The prApe1 protease protection was performed as previously described (58). Briefly, 50 ml cultures were grown to late-log phase (A_600_ 0,7–1,0) in YPD and subsequently starved for 45 min in SD-N. Cells were harvested, treated in 100 mM Pipes, pH 9.6, 10 mM DTT for 10 min at 30 °C, and finally converted into spheroplasts *via* treatment in 1.2 M sorbitol in SD-N with 5 mg lytic enzyme for 30 min at 30 °C. The spheroplasts were carefully pelleted and then subjected to osmotic lysis in 1 ml PS200 lysis buffer (20 mm Pipes, pH 6.8, 200 mm sorbitol, 5 mm MgCl_2_) containing 1 mM PMSF and 1× cOmplete protease inhibitor mix (Roche). After preclearing at 500*g* for 5 min at 4 °C, the lysed spheroplasts were centrifuged at 13,000*g* for 15 min at 4 °C. The supernatant was discarded and the pellet resuspended in PS200 buffer without any protease inhibitors and incubated in the presence or absence of proteinase K (100 μg/ml) and 0.4% Triton X-100, for 20 min on ice, followed by TCA precipitation, acetone wash, and immunoblot with an anti-Ape1 polyclonal antibody (2).

### APEX2-based proximity-dependent biotin labeling

One hundred A_600_ equivalents of cells were harvested by centrifugation at 3200*g* for 2 min at 4 °C. Cells were then prepared and processed for APEX2 proximity-dependent biotin labeling using a modified version of a protocol previously described (51). Briefly, cells were resuspended in 1 ml of freezing buffer (15% glycerol, 150 mM potassium acetate, 2 mM magnesium acetate, 20 mM Hepes/NaOH pH 7.2, 1% glucose), flash frozen in liquid nitrogen to semipermeabilize them, and stored at −80 °C for at least 1 day. Cells were next thawed on ice, centrifuged at 16′200*g* for 1 min at 4 °C, and upon discarding the supernatant, resuspended in 1 ml of freezing buffer at room temperature to which 0.5 mM biotin phenol was added. After gentle mixing, 1 mM H_2_O_2_ was added, and cells were gently mixed again. After 1 min incubation at room temperature, cells were mixed with 1 ml of ice-cold quenching buffer (PBS, 10 mM sodium ascorbate, 10 mM NaN_3_, 5 mM Trolox) to stop the reaction. To remove the excess BP and phenoxyl radicals, cells were washed five times with 1 ml of ice-cold quenching buffer by centrifugation at 16′200 rpm for 1 min at 4 °C. Cell pellets were finally flash frozen in liquid nitrogen and stored at −80 °C for subsequent proteomics analysis.

### Biotinylated proteins enrichment

Cell pellets were resuspended in lysis buffer (5% SDS, 50 mM Tris–HCl, pH 9, 100 mM DTT, 10 mM sodium ascorbate, 10 mM NaN_3_, 5 mM Trolox, 1 mM PMSF, and 1× cOmplete EDTA-free protease inhibitor mix) before to add glass beads, heating at 55 °C for 10 min under agitation (700 rpm) using a thermo-shaker (VWR Thermal Shake Touch), and vortexing 1 min at room temperature. The heating and vortexing was repeated two more times. Lysates were diluted in RIPA buffer (50 mM Tris–HCl, pH 7.5, 150 mM NaCl, 0.1% SDS, 0.5% sodium deoxycholate, 1% Triton X-100, 10 mM sodium ascorbate, 10 mM NaN_3_, 5 mM Trolox, 1 mM PMSF, and 1× cOmplete EDTA-free protease inhibitor mix).

Cleared lysates were loaded onto 100 μl streptavidin beads (High Capacity Streptavidin agarose, Thermo Fisher Scientific) prewashed with the lysis buffer and incubated overnight at 4 °C. Beads were successively washed with a solution of 1 M KCl, 0.1 M Na_2_CO_3_, 2 M urea, 50 mM Tris–HCl, pH 7.5. Proteins on beads were reduced with 1 mM DTT at room temperature for 15 min and alkylated with 5 mM iodoacetamide in the dark for 15 min. Then, 2 μg of trypsin (Promega) were added to each sample for overnight digestion at 37 °C. Supernatants were collected, acidified with 50% TFA, and desalted on 200 μl tips self-packed with a C18 disc (Thermo Fisher Scientific). Eluates were dried in a SpeedVac before being resuspend in 20 μl of 0.1% formic acid in water.

### Proteomic analysis

Liquid Chromatography with tandem mass spectrometry measurements were performed on a nano EasyLC (Thermo Fisher Scientific) coupled to a Q Exactive HF-X mass spectrometer (Thermo Fisher Scientific). Peptides were separated on a fused silica column (I.D. 75 μm, New Objective, self-packed with ReproSil-Pur 120 C18-AQ, 1.9 μm (Dr Maisch) to a length of 20 cm) using a gradient of A (0.1% formic acid in water) and B (0.1% formic acid in 80% acetonitrile in water) from 5% B to 30 over 85 min at a 250 nl/min flow rate.

The mass spectrometer was operated in data-independent acquisition mode with MS scans acquired at a resolution of 120k covering a m/z range from 370 to 1200, followed by 35 consecutive MS/MS scan windows of 24 m/z acquired at a 30k resolution with 1 m/z overlap. Raw data were analyzed using Spectronaut version 16.2.220903.53000 (89), searched against *S. cerevisiae* proteome and common contaminants (Uniprot, March 2016). Pulsar search was performed allowing for a maximum of three missed cleavages. Cysteine carbamidomethylation was set as fixed modification. Protein N-terminal acetylation and methionine oxidation were set as variable modifications. DIA analysis crossrun normalization was turned off. When not mentioned otherwise, all other settings were left to default BGS factory settings.

### Proteomic data analysis

Protein quantity values were log2 transformed, and samples were median normalized. Missing values in the control samples were imputed with random values drawn from a normal distribution of a mean 1.8 lower than the sample distribution and an SD of 0.3. Student’s t tests were performed in Perseus, and correction for multiple testing was done using random permutations (90).

### Subcellular fractionation

Separation of membranes present in low-speed supernatant fractions on sucrose gradients were performed as follows. One hundred A_600_ equivalents of spheroplasts were resuspended in 750 μl of ice-cold hypo-osmotic buffer (50 mM Tris–HCl, pH 7.5, 200 mM sorbitol, and 1 mM EDTA) containing freshly added cOmplete Protease Inhibitor Cocktail (Roche) and 1 mM PMSF, before to be lysed by Dounce homogenization. Cell lysates were centrifuged twice at 500 g for 5 min to remove cell debris. Then, 700 μl of the supernatant fractions (T) were subjected to centrifugation at 13,000 g for 15 min to be separated into supernatant (S13) and pellet (P13) fractions. Subsequently, 400 μl of S13 fraction were subsequently loaded on the top of sucrose step gradient (100 μl 22%, 100 μl 27%,150 μl 34%, 150 μl 37%, 150 μl 40%, 150 μl 45%, 150 μl 50%, and 100 μl 60% sucrose) in the gradient buffer (50 mM Tris–HCl, pH 7.5, 200 mM sorbitol, and 1 mM EDTA) and centrifuged at 190,000*g* for 8 h (TLS-55 rotor, Optima TLX Preparative Ultracentrifuge, Beckman Coulter). The top 400 μl were collected for the first fraction, and 13 fractions of 75 μl were then collected. While 20 μl were mixed with 6.7 μl of 4 × sample buffer and heated at 37 °C for 10 min, the rest was precipitated with 10% TCA and after washing with ice-cold acetone, resuspended in 55 μl of sample buffer and boiled at 95 °C for 10 min. Samples were finally resolved by SDS-PAGE followed by Western blot using antibodies or serum against MYC (Santa Cruz), HA (Cell Signaling Technology), Pma1 (EnCor Biotech), Tlg2 (91), and Pgk1. The samples heated at 37 °C were used for the detection of Atg9, whereas the ones boiled at 95 °C for the analysis of the other proteins.

### Fluorescence microscopy and image analysis

Cells were imaged in an Olympus IX-71 inverted microscope equipped with 100× NA 1,49 or 60× NA 1,40 objectives, sCMOS camera (PCO), InsightSSI illumination system, DAPI-, FITC-, mCherry-, YFP-, and Cy5-filters (Applied Precision). Z-stacks of 3 μm depth with 350 nm step size were recorded for each channel followed by subsequent deconvolution using the SoftWoRx software (Applied Precision). Vacuoles were labeled by incubating cells with 50 μM 7-amino-4-chloromethylcoumarin for 10 min before imaging.

The number of fluorescent Atg1, Atg8, Atg9, and Atg16 puncta per cell was quantified by segmentation of yeast cell boundaries in brightfield or digital image contrast images using YeastSpotter (92) and detection of fluorescent puncta per cell on sum projections of deconvolved image stacks as previously described (37) or using a Cellprofiler workflow adjusted for each fluorescently tagged construct (93). BiFC experiments were quantified by manual counting in ImageJ (National Institutes of Health).

### Electron microscopy

Electron microscopy examinations were performed and quantified as previously described (94).

### Statistical analysis

Statistical significance of Pho8Δ60 measurements was calculated using one-way ANOVA followed by Dunnett’s multiple comparisons test, and comparison of fluorescence imaging data was calculated using one-way ANOVA test and Dunn’s multiple comparisons test in Prism 8.4.2 (GraphPad Software). *p* values below ∗<0.015, ∗∗<0.0015 and ∗∗∗<0.0001 were considered significant.

## Data availability

All the data described are contained within the manuscript.

## Supporting information

This article contains supporting information.

## Conflict of interest

The authors declare no conflicts of interest in regard to this article.

## References

[1] Nakatogawa H. (2020). Mechanisms governing autophagosome biogenesis. Nat. Rev. Mol. Cell Biol..

[2] Mari M., Griffith J., Rieter E., Krishnappa L., Klionsky D.J., Reggiori F. (2010). An Atg9-containing compartment that functions in the early steps of autophagosome biogenesis. J. Cell Biol..

[3] Yamamoto H., Kakuta S., Watanabe T.M., Kitamura A., Sekito T., Kondo-Kakuta C. (2012). Atg9 vesicles are an important membrane source during early steps of autophagosome formation. J. Cell Biol..

[4] Orsi A., Razi M., Dooley H.C., Robinson D., Weston A.E., Collinson L.M. (2012). Dynamic and transient interactions of Atg9 with autophagosomes, but not membrane integration, are required for autophagy. Mol. Biol. Cell.

[5] Young A.R.J., Chan E.Y.W., Hu X.W., Köchl R., Crawshaw S.G., High S. (2006). Starvation and ULK1-dependent cycling of mammalian Atg9 between the TGN and endosomes. J. Cell Sci..

[6] Noda T., Kim J., Huang W.-P., Baba M., Tokunaga C., Ohsumi Y. (2000). Apg9p/Cvt7p is an integral membrane protein required for transport vesicle formation in the Cvt and autophagy pathways. J. Cell Biol..

[7] Maeda S., Yamamoto H., Kinch L.N., Garza C.M., Takahashi S., Otomo C. (2020). Structure, lipid scrambling activity and role in autophagosome formation of ATG9A. Nat. Struct. Mol. Biol..

[8] Matoba K., Kotani T., Tsutsumi A., Tsuji T., Mori T., Noshiro D. (2020). Atg9 is a lipid scramblase that mediates autophagosomal membrane expansion. Nat. Struct. Mol. Biol..

[9] Chumpen Ramirez S., Gomez-Sanchez R., Verlhac P., Hardenberg R., Margheritis E., Cosentino K. (2022). Atg9 interactions *via* its transmembrane domains are required for phagophore expansion during autophagy. Autophagy.

[10] He C., Song H., Yorimitsu T., Monastyrska I., Yen W.-L., Legakis J.E. (2006). Recruitment of Atg9 to the preautophagosomal structure by Atg11 is essential for selective autophagy in budding yeast. J. Cell Biol..

[11] Sekito T., Kawamata T., Ichikawa R., Suzuki K., Ohsumi Y. (2009). Atg17 recruits Atg9 to organize the pre-autophagosomal structure. Genes Cells.

[12] Suzuki K., Akioka M., Kondo-Kakuta C., Yamamoto H., Ohsumi Y. (2013). Fine mapping of autophagy-related proteins during autophagosome formation in *Saccharomyces cerevisiae*. J. Cell Sci..

[13] Suzuki K., Kirisako T., Kamada Y., Mizushima N., Noda T., Ohsumi Y. (2001). The pre-autophagosomal structure organized by concerted functions of *APG* genes is essential for autophagosome formation. EMBO J..

[14] Graef M., Friedman J.R., Graham C., Babu M., Nunnari J. (2013). ER exit sites are physical and functional core autophagosome biogenesis components. Mol. Biol. Cell.

[15] Kim J., Huang W.-P., Stromhaug P.E., Klionsky D.J. (2002). Convergence of multiple autophagy and cytoplasm to vacuole targeting components to a perivacuolar membrane compartment prior to *de novo* vesicle formation. J. Biol. Chem..

[16] Gao J., Reggiori F., Ungermann C. (2018). A novel *in vitro* assay reveals SNARE topology and the role of Ykt6 in autophagosome fusion with vacuoles. J. Cell Biol..

[17] Cebollero E., van der Vaart A., Zhao M., Rieter E., Klionsky D.J., Helms J.B. (2012). Phosphatidylinositol-3-phosphate clearance plays a key role in autophagosome completion. Curr. Biol..

[18] Gallon M., Cullen P.J. (2015). Retromer and sorting nexins in endosomal sorting. Biochem. Soc. Trans..

[19] Ferguson S.M., De Camilli P. (2012). Dynamin, a membrane-remodelling GTPase. Nat. Rev. Mol. Cell Biol..

[20] Bohm J., Biancalana V., Dechene E.T., Bitoun M., Pierson C.R., Schaefer E. (2012). Mutation spectrum in the large GTPase dynamin 2, and genotype-phenotype correlation in autosomal dominant centronuclear myopathy. Hum. Mutat..

[21] Brown F.C., Collett M., Tremblay C.S., Rank G., De Camilli P., Booth C.J. (2017). Loss of Dynamin 2 GTPase function results in microcytic anaemia. Br. J. Haematol..

[22] Popovic D., Dikic I. (2014). TBC1D5 and the AP2 complex regulate ATG9 trafficking and initiation of autophagy. EMBO Rep..

[23] Zavodszky E., Seaman M.N., Moreau K., Jimenez-Sanchez M., Breusegem S.Y., Harbour M.E. (2014). Mutation in VPS35 associated with Parkinson's disease impairs WASH complex association and inhibits autophagy. Nat. Commun..

[24] Lamb C.A., Nuhlen S., Judith D., Frith D., Snijders A.P., Behrends C. (2016). TBC1D14 regulates autophagy *via* the TRAPP complex and ATG9 traffic. EMBO J..

[25] Shirahama-Noda K., Kira S., Yoshimori T., Noda T. (2013). TRAPPIII is responsible for vesicular transport from early endosomes to Golgi, facilitating Atg9 cycling in autophagy. J. Cell Sci..

[26] Lynch-Day M.A., Bhandari D., Menon S., Huang J., Cai H., Bartholomew C.R. (2010). Trs85 directs a Ypt1 GEF, TRAPPIII, to the phagophore to promote autophagy. Proc. Natl. Acad. Sci. U. S. A..

[27] Ravikumar B., Moreau K., Jahreiss L., Puri C., Rubinsztein D.C. (2010). Plasma membrane contributes to the formation of pre-autophagosomal structures. Nat. Cell Biol..

[28] Davies A.K., Itzhak D.N., Edgar J.R., Archuleta T.L., Hirst J., Jackson L.P. (2018). AP-4 vesicles contribute to spatial control of autophagy *via* RUSC-dependent peripheral delivery of ATG9A. Nat. Commun..

[29] Mattera R., Park S.Y., De Pace R., Guardia C.M., Bonifacino J.S. (2017). AP-4 mediates export of ATG9A from the trans-Golgi network to promote autophagosome formation. Proc. Natl. Acad. Sci. U. S. A..

[30] Longatti A., Lamb C.A., Razi M., Yoshimura S., Barr F.A., Tooze S.A. (2012). TBC1D14 regulates autophagosome formation *via* Rab11- and ULK1-positive recycling endosomes. J. Cell Biol..

[31] Knaevelsrud H., Soreng K., Raiborg C., Haberg K., Rasmuson F., Brech A. (2013). Membrane remodeling by the PX-BAR protein SNX18 promotes autophagosome formation. J. Cell Biol..

[32] Takahashi Y., Tsotakos N., Liu Y., Young M.M., Serfass J., Tang Z. (2016). The Bif-1-Dynamin 2 membrane fission machinery regulates Atg9-containing vesicle generation at the Rab11-positive reservoirs. Oncotarget.

[33] Soreng K., Munson M.J., Lamb C.A., Bjorndal G.T., Pankiv S., Carlsson S.R. (2018). SNX18 regulates ATG9A trafficking from recycling endosomes by recruiting Dynamin-2. EMBO Rep..

[34] Nice D.C., Sato T.K., Stromhaug P.E., Emr S.D., Klionsky D.J. (2002). Cooperative binding of the cytoplasm to vacuole targeting pathway proteins, Cvt13 and Cvt20, to PtdIns(3)P at the pre-autophagosomal structure is required for selective autophagy. J. Biol. Chem..

[35] Guimaraes R.S., Delorme-Axford E., Klionsky D.J., Reggiori F. (2015). Assays for the biochemical and ultrastructural measurement of selective and nonselective types of autophagy in the yeast Saccharomyces cerevisiae. Methods.

[36] Dengjel J., Hoyer-Hansen M., Nielsen M.O., Eisenberg T., Harder L.M., Schandorff S. (2012). Identification of autophagosome-associated proteins and regulators by quantitative proteomic analysis and genetic screens. Mol. Cell Proteomics.

[37] Arlt H., Reggiori F., Ungermann C. (2015). Retromer and the dynamin Vps1 cooperate in the retrieval of transmembrane proteins from vacuoles. J. Cell Sci..

[38] Chi R.J., Liu J., West M., Wang J., Odorizzi G., Burd C.G. (2014). Fission of SNX-BAR-coated endosomal retrograde transport carriers is promoted by the dynamin-related protein Vps1. J. Cell Biol..

[39] Kamada Y., Funakoshi T., Shintani T., Nagano K., Ohsumi M., Ohsumi Y. (2000). Tor-mediated induction of autophagy *via* an Apg1 protein kinase complex. J. Cell Biol..

[40] Nothwehr S.F., Conibear E., Stevens T.H. (1995). Golgi and vacuolar membrane proteins reach the vacuole in vps1 mutant yeast cells *via* the plasma membrane. J. Cell Biol..

[41] Kirisako T., Baba M., Ishihara N., Miyazawa K., Ohsumi M., Yoshimori T. (1999). Formation process of autophagosome is traced with Apg8/Aut7p in yeast. J. Cell Biol..

[42] Mari M., Reggiori F. (2010). Atg9 reservoirs, a new organelle of the yeast endomembrane system?. Autophagy.

[43] Shintani T., Reggiori F. (2008). Fluorescence microscopy-based assays for monitoring yeast Atg protein trafficking. Met. Enzymol..

[44] Reggiori F., Tucker K.A., Stromhaug P.E., Klionsky D.J. (2004). The Atg1-Atg13 complex regulates Atg9 and Atg23 retrieval transport from the pre-autophagosomal structure. Dev. Cell.

[45] Marquardt L., Taylor M., Kramer F., Schmitt K., Braus G.H., Valerius O. (2022). Vacuole fragmentation depends on a novel Atg18-containing retromer-complex. Autophagy.

[46] Palmer S.E., Smaczynska-de R., II, Marklew C.J., Allwood E.G., Mishra R., Johnson S. (2015). A dynamin-actin interaction is required for vesicle scission during endocytosis in yeast. Curr. Biol..

[47] Chen X., Zaro J.L., Shen W.C. (2013). Fusion protein linkers: property, design and functionality. Adv. Drug Deliv. Rev..

[48] Sung M.K., Huh W.K. (2007). Bimolecular fluorescence complementation analysis system for *in vivo* detection of protein-protein interaction in Saccharomyces cerevisiae. Yeast.

[49] Arlt H., Auffarth K., Kurre R., Lisse D., Piehler J., Ungermann C. (2015). Spatiotemporal dynamics of membrane remodeling and fusion proteins during endocytic transport. Mol. Biol. Cell.

[50] Losev E., Reinke C.A., Jellen J., Strongin D.E., Bevis B.J., Glick B.S. (2006). Golgi maturation visualized in living yeast. Nature.

[51] Singer-Kruger B., Frohlich T., Franz-Wachtel M., Nalpas N., Macek B., Jansen R.P. (2020). APEX2-mediated proximity labeling resolves protein networks in *Saccharomyces cerevisiae* cells. FEBS J..

[52] Legakis J.E., Yen W.-L., Klionsky D.J. (2007). A cycling protein complex required for selective autophagy. Autophagy.

[53] Reggiori F., Shintani T., Nair U., Klionsky D.J. (2005). Atg9 cycles between mitochondria and the pre-autophagosomal structure in yeasts. Autophagy.

[54] Lynch-Day M.A., Klionsky D.J. (2010). The Cvt pathway as a model for selective autophagy. FEBS Lett..

[55] Darsow T., Rieder S.E., Emr S.D. (1997). A multispecificity syntaxin homologue, Vam3p, essential for autophagic and biosynthetic protein transport to the vacuole. J. Cell Biol..

[56] Bas L., Papinski D., Licheva M., Torggler R., Rohringer S., Schuschnig M. (2018). Reconstitution reveals Ykt6 as the autophagosomal SNARE in autophagosome-vacuole fusion. J. Cell Biol.

[57] Matsuura A., Tsukada M., Wada Y., Ohsumi Y. (1997). Apg1p, a novel protein kinase required for the autophagic process in *Saccharomyces cerevisiae*. Gene.

[58] Nair U., Thumm M., Klionsky D.J., Krick R. (2011). GFP-Atg8 protease protection as a tool to monitor autophagosome biogenesis. Autophagy.

[59] Varlakhanova N.V., Alvarez F.J.D., Brady T.M., Tornabene B.A., Hosford C.J., Chappie J.S. (2018). Structures of the fungal dynamin-related protein Vps1 reveal a unique, open helical architecture. J. Cell Biol..

[60] Moustaq L., Smaczynska-de R., II Palmer S.E., Marklew C.J., Ayscough K.R. (2016). Insights into dynamin-associated disorders through analysis of equivalent mutations in the yeast dynamin Vps1. Microb. Cell.

[61] Smaczynska-de R., Marklew C.J., Allwood E.G., Palmer S.E., Booth W.I., Mishra R. (2015). Phosphorylation regulates the endocytic function of the yeast dynamin-related protein Vps1. Mol. Cell Biol..

[62] Song B.D., Yarar D., Schmid S.L. (2004). An assembly-incompetent mutant establishes a requirement for dynamin self-assembly in clathrin-mediated endocytosis *in vivo*. Mol. Biol. Cell.

[63] Smaczynska-de R., II, Allwood E.G., Aghamohammadzadeh S., Hettema E.H., Goldberg M.W., Ayscough K.R. (2010). A role for the dynamin-like protein Vps1 during endocytosis in yeast. J. Cell Sci..

[64] Bitoun M., Bevilacqua J.A., Eymard B., Prudhon B., Fardeau M., Guicheney P. (2009). A new centronuclear myopathy phenotype due to a novel dynamin 2 mutation. Neurology.

[65] Peters C., Baars T.L., Buhler S., Mayer A. (2004). Mutual control of membrane fission and fusion proteins. Cell.

[66] Vater C.A., Raymond C.K., Ekena K., Howald-Stevenson I., Stevens T.H. (1992). The VPS1 protein, a homolog of dynamin required for vacuolar protein sorting in *Saccharomyces cerevisiae*, is a GTPase with two functionally separable domains. J. Cell Biol..

[67] Alpadi K., Kulkarni A., Namjoshi S., Srinivasan S., Sippel K.H., Ayscough K. (2013). Dynamin-SNARE interactions control trans-SNARE formation in intracellular membrane fusion. Nat. Commun..

[68] Takahashi Y., Meyerkord C.L., Hori T., Runkle K., Fox T.E., Kester M. (2011). Bif-1 regulates Atg9 trafficking by mediating the fission of Golgi membranes during autophagy. Autophagy.

[69] Anton Z., Betin V.M.S., Simonetti B., Traer C.J., Attar N., Cullen P.J. (2020). A heterodimeric SNX4:SNX7 SNX-BAR autophagy complex coordinates ATG9A trafficking for efficient autophagosome assembly. J. Cell Sci..

[70] Courtellemont T., De Leo M.G., Gopaldass N., Mayer A. (2022). Crop: a retromer-PROPPIN complex mediating membrane fission in the endo-lysosomal system. EMBO J..

[71] Ohashi Y., Munro S. (2010). Membrane delivery to the yeast autophagosome from the Golgi-endosomal system. Mol. Biol. Cell.

[72] Hollenstein D.M., Gomez-Sanchez R., Ciftci A., Kriegenburg F., Mari M., Torggler R. (2019). Vac8 spatially confines autophagosome formation at the vacuole in *S. cerevisiae*. J. Cell Sci..

[73] Jin M., He D., Backues S.K., Freeberg M.A., Liu X., Kim J.K. (2014). Transcriptional regulation by Pho23 modulates the frequency of autophagosome formation. Curr. Biol..

[74] Puri C., Manni M.M., Vicinanza M., Hilcenko C., Zhu Y., Runwal G. (2020). A DNM2 centronuclear myopathy mutation reveals a link between recycling endosome scission and autophagy. Dev. Cell.

[75] Menzies F.M., Fleming A., Caricasole A., Bento C.F., Andrews S.P., Ashkenazi A. (2017). Autophagy and neurodegeneration: pathogenic mechanisms and therapeutic opportunities. Neuron.

[76] Stamatakou E., Wrobel L., Hill S.M., Puri C., Son S.M., Fujimaki M. (2020). Mendelian neurodegenerative disease genes involved in autophagy. Cell Discov..

[77] van Beek N., Klionsky D.J., Reggiori F. (2018). Genetic aberrations in macroautophagy genes leading to diseases. Biochim. Biophys. Acta.

[78] Durieux A.C., Prudhon B., Guicheney P., Bitoun M. (2010). Dynamin 2 and human diseases. J. Mol. Med. (Berl).

[79] Janke C., Magiera M.M., Rathfelder N., Taxis C., Reber S., Maekawa H. (2004). A versatile toolbox for PCR-based tagging of yeast genes: new fluorescent proteins, more markers and promoter substitution cassettes. Yeast.

[80] Longtine M.S., McKenzie A., Demarini D.J., Shah N.G., Wach A., Brachat A. (1998). Additional modules for versatile and economical PCR-based gene deletion and modification in *Saccharomyces cerevisiae*. Yeast.

[81] Kim J., Huang W.-P., Klionsky D.J. (2001). Membrane recruitment of Aut7p in the autophagy and cytoplasm to vacuole targeting pathways requires Aut1p, Aut2p, and the autophagy conjugation complex. J. Cell Biol..

[82] Sikorski R.S., Hieter P. (1989). A system of shuttle vectors and yeast host strains designed for efficient manipulation of DNA in *Saccharomyces cerevisiae*. Genetics.

[83] Lewis M.J., Nichols B.J., Prescianotto-Baschong C., Riezman H., Pelham H.R. (2000). Specific retrieval of the exocytic SNARE Snc1p from early yeast endosomes. Mol. Biol. Cell.

[84] Palmer S.E., Smaczynska-de R., II, Marklew C.J., Allwood E.G., Mishra R., Goldberg M.W. (2015). A Charge Swap mutation E461K in the yeast dynamin Vps1 reduces endocytic invagination. Commun. Integr. Biol..

[85] Mishra R., Smaczynska-de R., Goldberg M.W., Ayscough K.R. (2011). Expression of Vps1 I649K a self-assembly defective yeast dynamin, leads to formation of extended endocytic invaginations. Commun. Integr. Biol..

[86] Abreu S., Kriegenburg F., Gomez-Sanchez R., Mari M., Sanchez-Wandelmer J., Skytte Rasmussen M. (2017). Conserved Atg8 recognition sites mediate Atg4 association with autophagosomal membranes and Atg8 deconjugation. EMBO Rep..

[87] Sanchez-Wandelmer J., Kriegenburg F., Rohringer S., Schuschnig M., Gomez-Sanchez R., Zens B. (2017). Atg4 proteolytic activity can be inhibited by Atg1 phosphorylation. Nat. Commun..

[88] Whyte J.R., Munro S. (2001). A yeast homolog of the mammalian mannose 6-phosphate receptors contributes to the sorting of vacuolar hydrolases. Curr. Biol..

[89] Bruderer R., Bernhardt O.M., Gandhi T., Miladinovic S.M., Cheng L.Y., Messner S. (2015). Extending the limits of quantitative proteome profiling with data-independent acquisition and application to acetaminophen-treated three-dimensional liver microtissues. Mol. Cell Proteomics.

[90] Tyanova S., Temu T., Sinitcyn P., Carlson A., Hein M.Y., Geiger T. (2016). The Perseus computational platform for comprehensive analysis of (prote)omics data. Nat. Met..

[91] Holthuis J.C., Nichols B.J., Dhruvakumar S., Pelham H.R.B. (1998). Two syntaxin homologues in the TGN/endosomal system of yeast. EMBO J..

[92] Lu A.X., Zarin T., Hsu I.S., Moses A.M. (2019). YeastSpotter: accurate and parameter-free web segmentation for microscopy images of yeast cells. Bioinformatics.

[93] Carpenter A.E., Jones T.R., Lamprecht M.R., Clarke C., Kang I.H., Friman O. (2006). CellProfiler: Image analysis software for identifying and quantifying cell phenotypes. Genome Biol..

[94] Griffith J., Mari M., De Maziere A., Reggiori F. (2008). A cryosectioning procedure for the ultrastructural analysis and the immunogold labelling of yeast *Saccharomyces cerevisiae*. Traffic.

[95] Marks B., Stowell M.H., Vallis Y., Mills I.G., Gibson A., Hopkins C.R. (2001). GTPase activity of dynamin and resulting conformation change are essential for endocytosis. Nature.

[96] Song B.D., Leonard M., Schmid S.L. (2004). Dynamin GTPase domain mutants that differentially affect GTP binding, GTP hydrolysis, and clathrin-mediated endocytosis. J. Biol. Chem..

[97] Narayanan R., Leonard M., Song B.D., Schmid S.L., Ramaswami M. (2005). An internal GAP domain negatively regulates presynaptic dynamin *in vivo*: a two-step model for dynamin function. J. Cell Biol..

[98] Damke H., Baba T., van der Bliek A.M., Schmid S.L. (1995). Clathrin-independent pinocytosis is induced in cells overexpressing a temperature-sensitive mutant of dynamin. J. Cell Biol.

[99] Sever S., Muhlberg A.B., Schmid S.L. (1999). Impairment of dynamin's GAP domain stimulates receptor-mediated endocytosis. Nature.

[100] Wang C.-W., Stromhaug P.E., Shima J., Klionsky D.J. (2002). The Ccz1-Mon1 protein complex is required for the late step of multiple vacuole delivery pathways. J. Biol. Chem..

[101] Robinson J.S., Klionsky D.J., Banta L.M., Emr S.D. (1988). Protein sorting in *Saccharomyces cerevisiae*: isolation of mutants defective in the delivery and processing of multiple vacuolar hydrolases. Mol. Cell Biol..

[102] Cheong H., Nair U., Geng J., Klionsky D.J. (2008). The Atg1 kinase complex is involved in the regulation of protein recruitment to initiate sequestering vesicle formation for nonspecific autophagy in *Saccharomyces cerevisiae*. Mol. Biol. Cell.

[103] Shintani T., Huang W.-P., Stromhaug P.E., Klionsky D.J. (2002). Mechanism of cargo selection in the cytoplasm to vacuole targeting pathway. Dev. Cell.

[104] Kanki T., Wang K., Baba M., Bartholomew C.R., Lynch-Day M.A., Du Z. (2009). A genomic screen for yeast mutants defective in selective mitochondria autophagy. Mol. Biol. Cell.

