# Supplementary figures and images for: The dynamin Vps1 mediates Atg9 transport to the sites of autophagosome formation
Source: J Biol Chem. 2023 Apr 14;299(5):104712. doi: 10.1016/j.jbc.2023.104712 (PMC10196871; doi:10.1016/j.jbc.2023.104712)

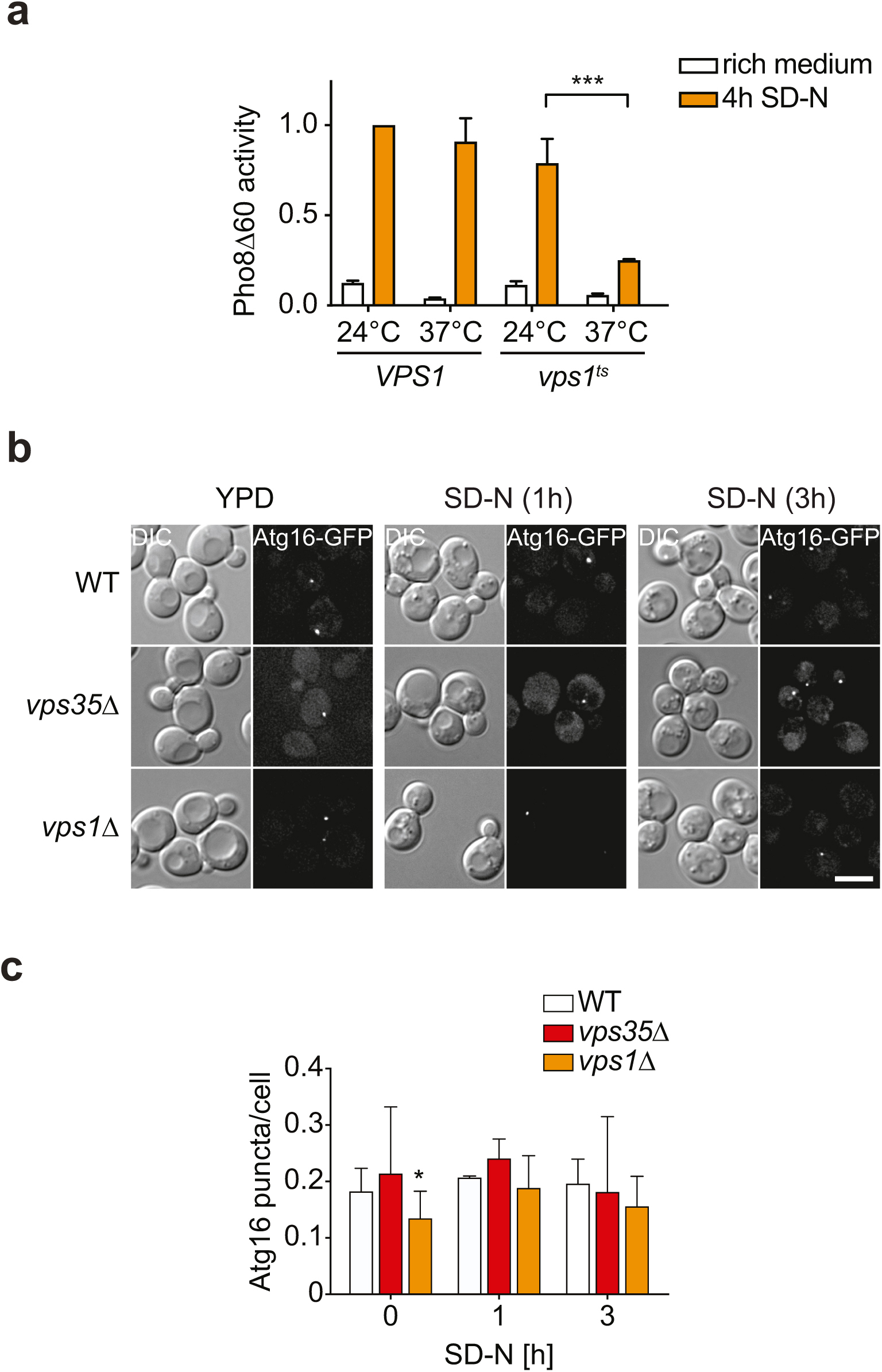

Supplement: Figure S1 [file figs1.jpg]

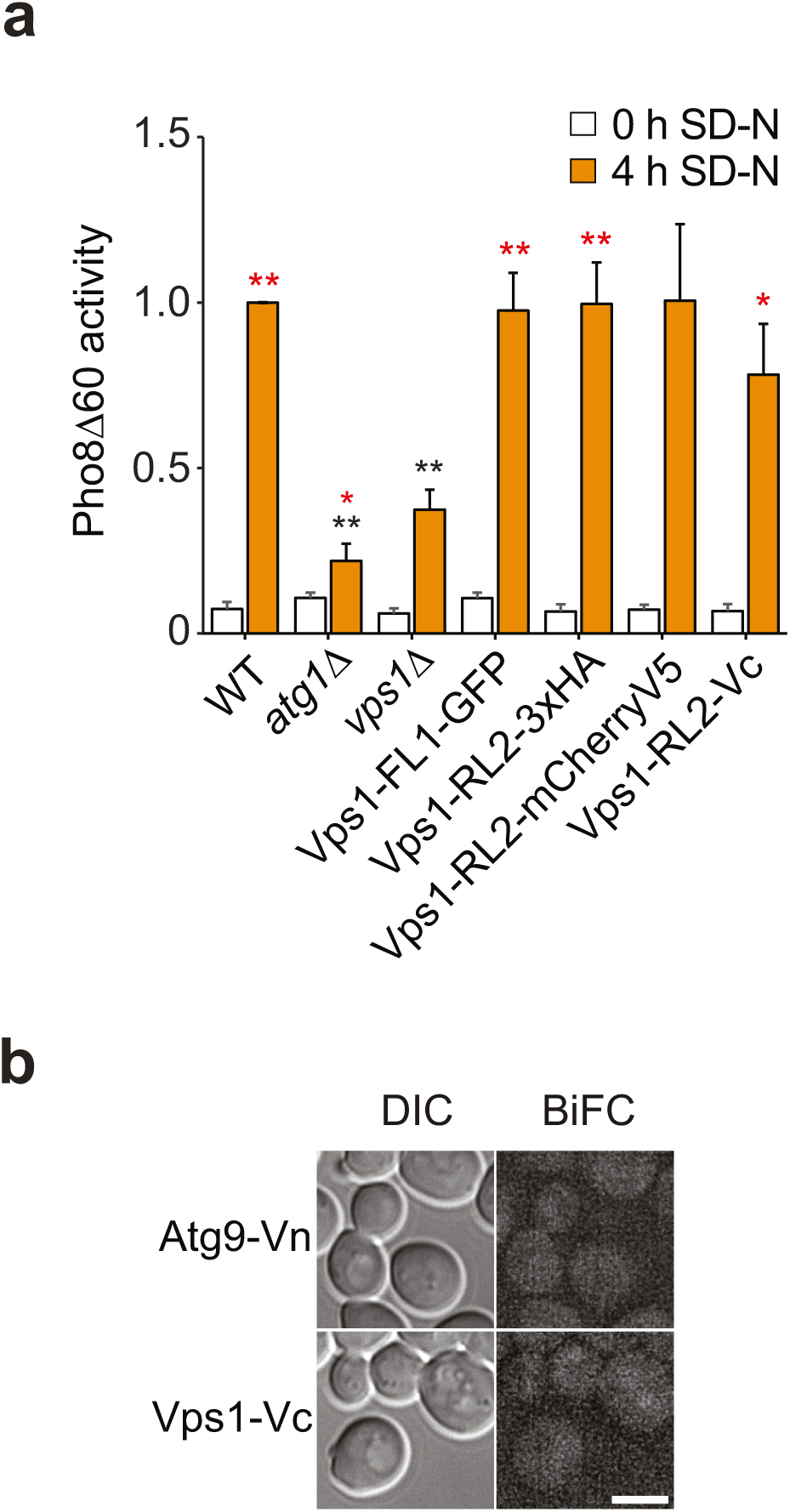

Supplement: Figure S2 [file figs2.jpg]

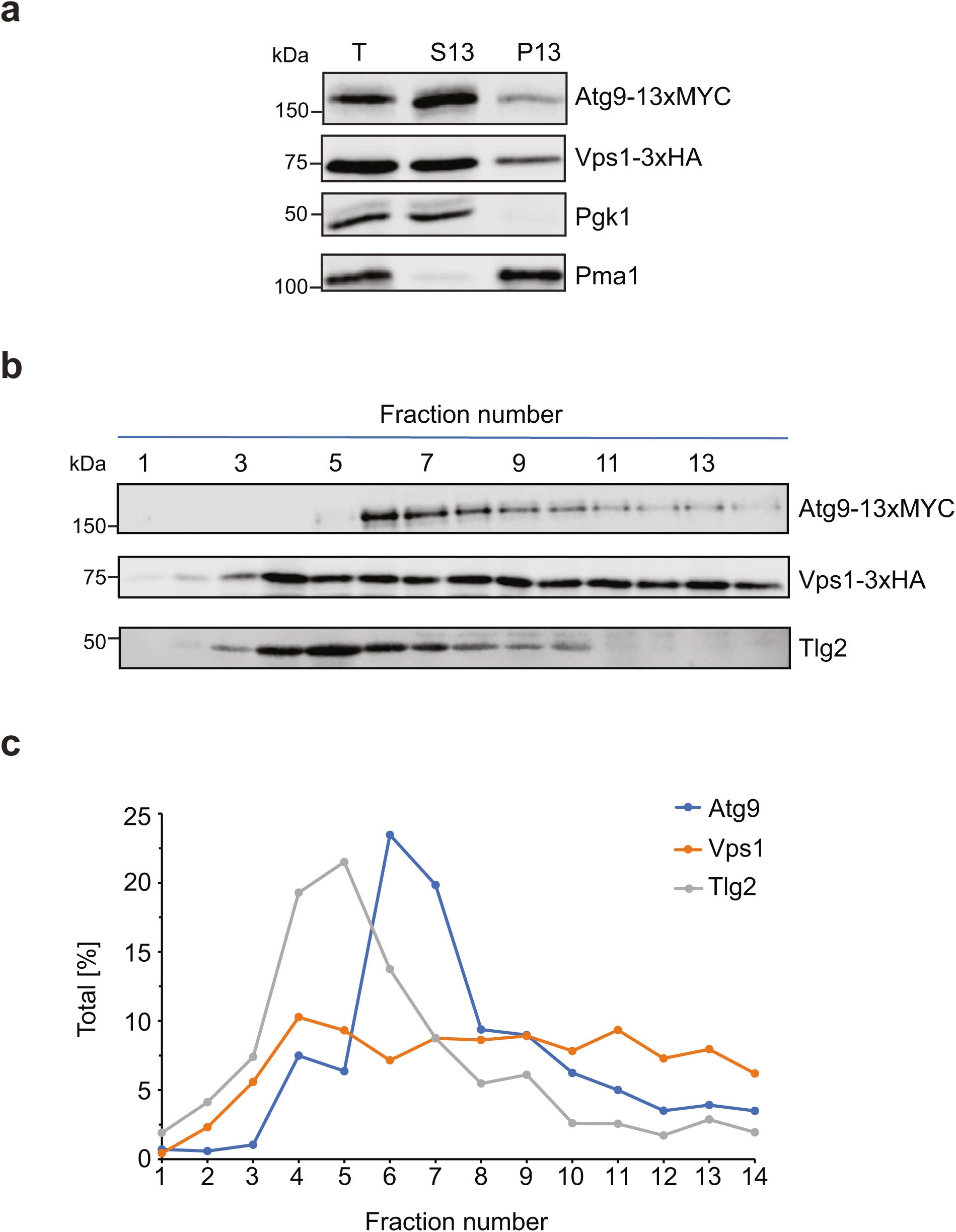

Supplement: Figure S3 [file figs3.jpg]

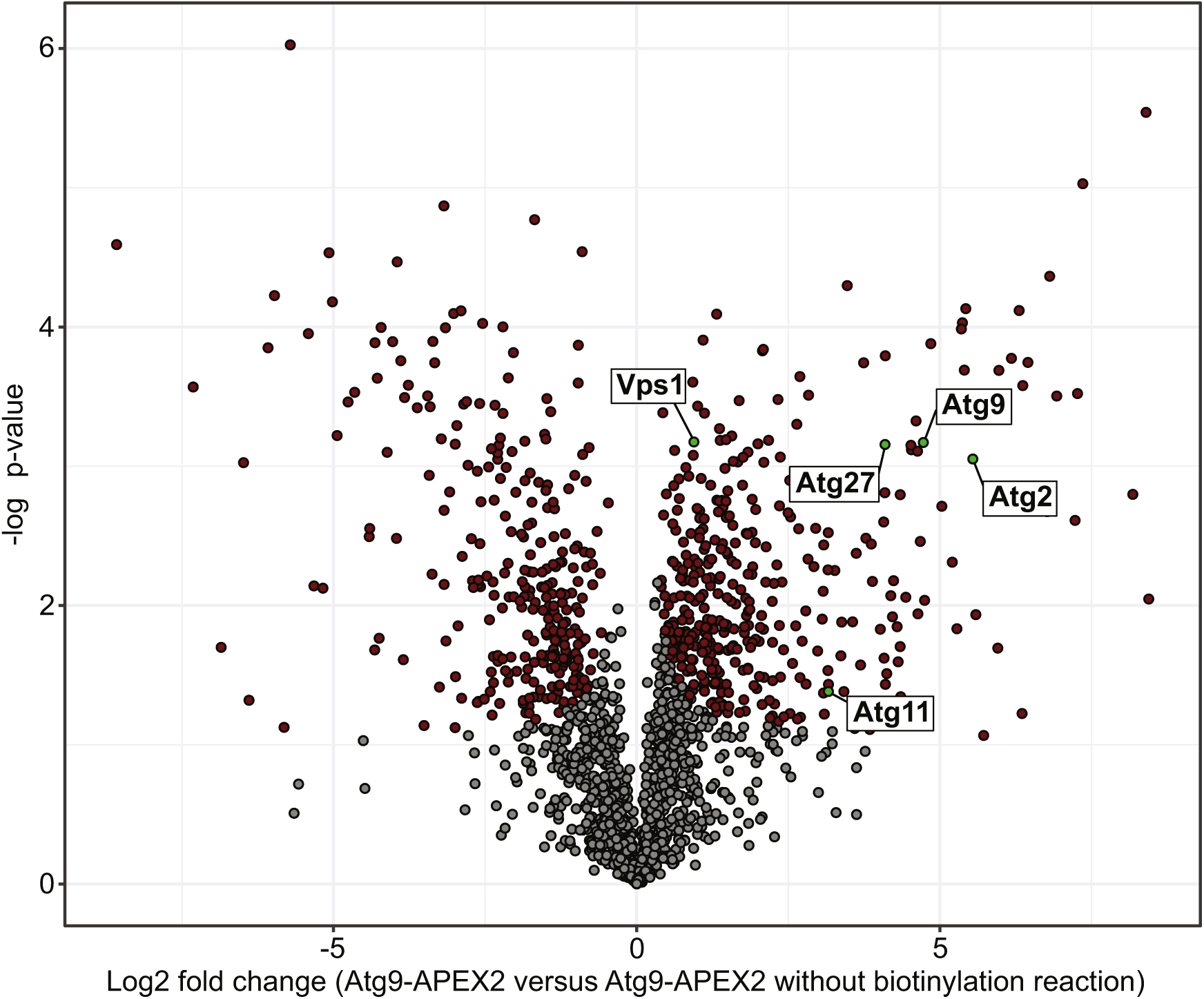

Supplement: Figure S4 [file figs4.jpg]

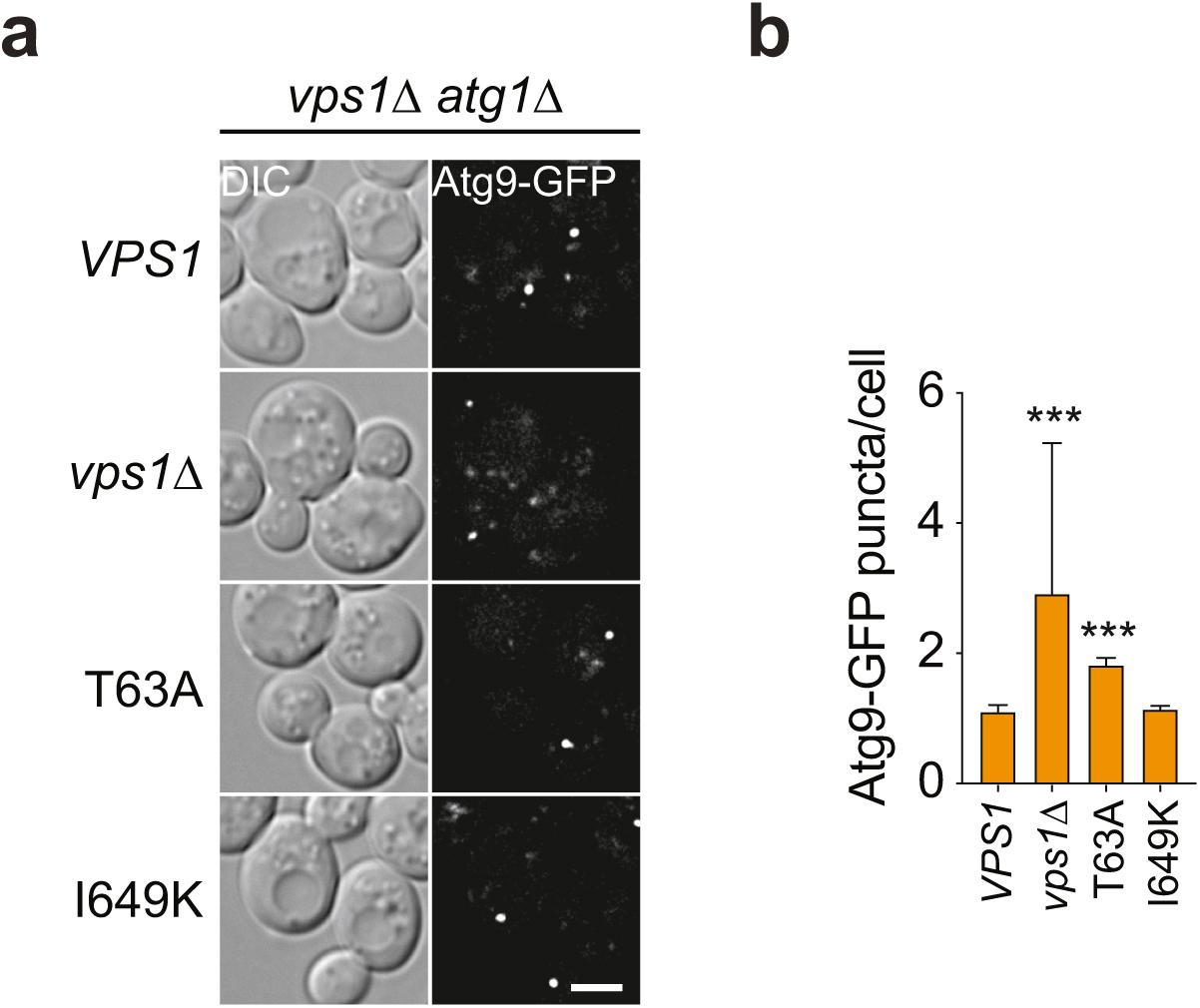

Supplement: Figure S5 [file figs5.jpg]

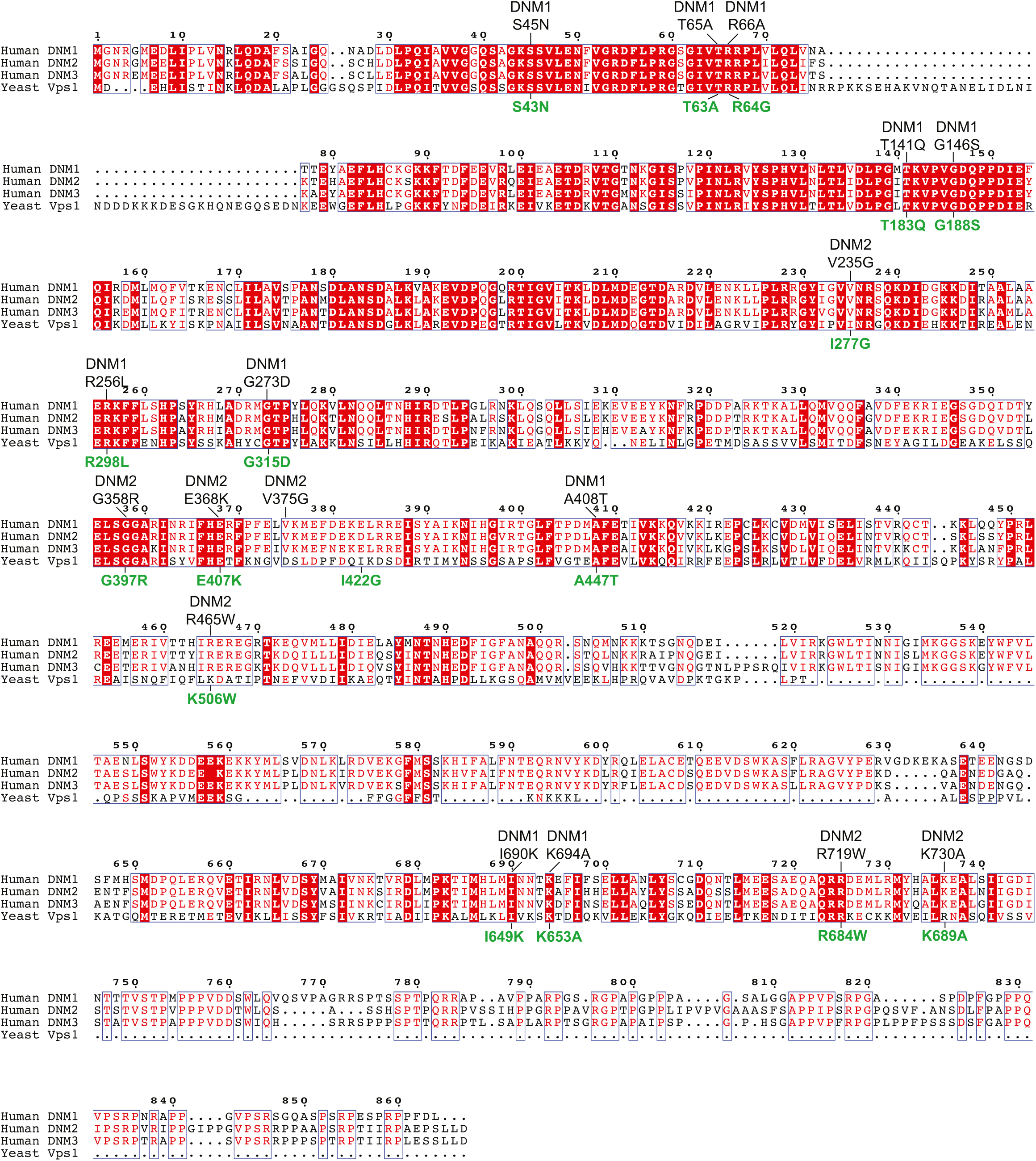

Supplement: Figure S6 [file figs6.jpg]

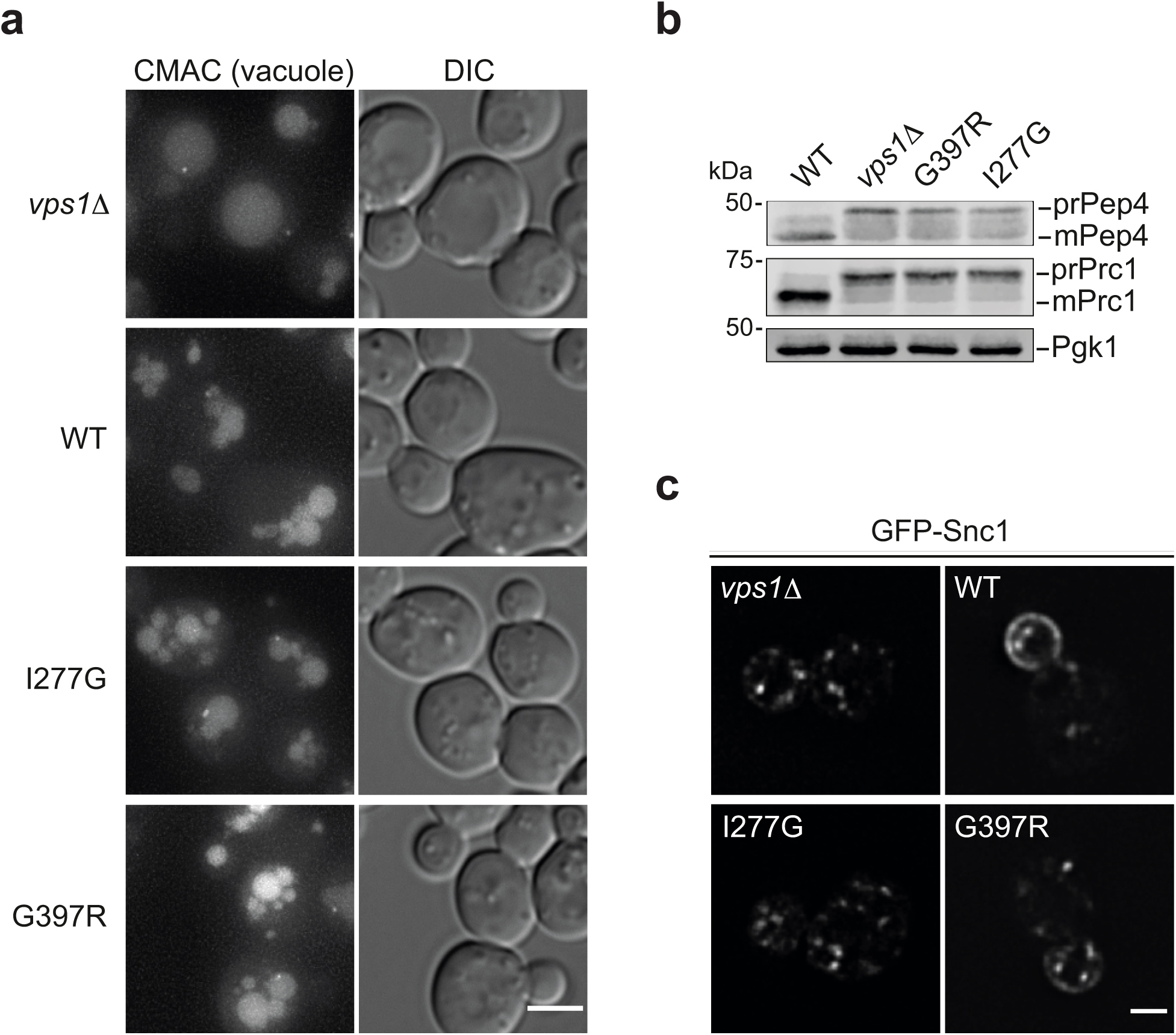

Supplement: Figure S7 [file figs7.jpg]
